# Least-squares methods for identifying biochemical regulatory networks from noisy measurements

**DOI:** 10.1186/1471-2105-8-8

**Published:** 2007-01-10

**Authors:** Jongrae Kim, Declan G Bates, Ian Postlethwaite, Pat Heslop-Harrison, Kwang-Hyun Cho

**Affiliations:** 1 Department of Engineering, University of Leicester, Leicester, LE1 7RH, UK; 2 Department of Biology, University of Leicester, Leicester, LE1 7RH, UK; 3 College of Medicine, Seoul National University, Jongno-gu, Seoul, 110-799, Korea; 4 Bio-MAX Institute, Seoul National University, Gwanak-gu, Seoul, 151-818, Korea

## Abstract

**Background:**

We consider the problem of identifying the dynamic interactions in biochemical networks from noisy experimental data. Typically, approaches for solving this problem make use of an estimation algorithm such as the well-known linear Least-Squares (LS) estimation technique. We demonstrate that when time-series measurements are corrupted by white noise and/or drift noise, more accurate and reliable identification of network interactions can be achieved by employing an estimation algorithm known as Constrained Total Least Squares (CTLS). The Total Least Squares (TLS) technique is a generalised least squares method to solve an overdetermined set of equations whose coefficients are noisy. The CTLS is a natural extension of TLS to the case where the noise components of the coefficients are correlated, as is usually the case with time-series measurements of concentrations and expression profiles in gene networks.

**Results:**

The superior performance of the CTLS method in identifying network interactions is demonstrated on three examples: a genetic network containing four genes, a network describing p53 activity and *mdm2 *messenger RNA interactions, and a recently proposed kinetic model for interleukin (IL)-6 and (IL)-12b messenger RNA expression as a function of ATF3 and NF-*κ*B promoter binding. For the first example, the CTLS significantly reduces the errors in the estimation of the Jacobian for the gene network. For the second, the CTLS reduces the errors from the measurements that are corrupted by white noise and the effect of neglected kinetics. For the third, it allows the correct identification, from noisy data, of the negative regulation of (IL)-6 and (IL)-12b by ATF3.

**Conclusion:**

The significant improvements in performance demonstrated by the CTLS method under the wide range of conditions tested here, including different levels and types of measurement noise and different numbers of data points, suggests that its application will enable more accurate and reliable identification and modelling of biochemical networks.

## Background

A key objective of Systems Biology research is to move from a qualitative to a quantitative understanding of cellular signalling and gene networks. Motivated by recent advances in high-throughput genomics and proteomics analysis, and the resulting explosive growth in the amount of data available for analysis, much effort is currently focused on developing reliable methods for inferring the structural and functional organisation of biochemical networks from data obtained by time-series measurements – see for example [1-6] and references therein.

Interactions between components of biological networks can conveniently be represented by weighted, directed graphs, where the nodes correspond to the biochemical components, and the edges, represented as arrows with weights attached, indicate the direct quantitative effect that a change in one component has on another component [1]. The weights are, in general, nonlinear functions that represent often largely unknown reaction kinetics, and it is therefore not usually practical to directly determine these weights from experimental data. This is particularly the case for gene networks whose structures are poorly understood in general, even qualitatively. In such cases, a useful approach is to consider the biochemical network behaviour about some steady-state, and assume that it behaves linearly for small deviations from this steady-state [2,3]. With this assumption, the network weights become constants, quantifying the reactions between the components in the neighbourhood of the steady-state. An interaction matrix, known as the Jacobian, is then obtained by grouping the constant weights into a matrix.

Several different approaches for determining the Jacobian of a network from time-series data have recently appeared in the literature [1-6]. A common feature of all these approaches is that the network is perturbed in some way, and then data are collected from time-series measurements of one or more components of the network. In [1], an approach was proposed which can handle very general types of system perturbations, such as gene knockouts and inhibitor additions. For these types of perturbations, the exact size, as well as the direct effect of the perturbations will be largely unknown, and therefore the method also allows the determination of the perturbation itself from the data. Another advantage of the approach of [1] is that the effect of unsteady-state initial conditions can be treated as an unknown perturbation and hence also estimated from the data. This removes the requirement for the system to be in a steady-state with known activities and concentrations when the perturbation is applied.

Another common feature of almost all the approaches for reverse engineering biomolecular networks so far proposed in the literature is that they employ some estimation algorithm to infer network structure from the measurement data. In [1], for example, the basis of the method for simultaneous estimation of the system states and parameter perturbations is a linear least-squares algorithm. A significant limitation of most such algorithms is that they do not take account of the noise that is inevitably present in the measurement data. Indeed, in the results presented in [1], it was observed that significant levels of noise in the measurement data could lead to quite large errors in the estimated Jacobian matrix.

In data from most biological experiments, the error associated with each measurement is substantial. The amount of measurement noise is often poorly defined but arises from 1) errors inherent in the measurement technique; 2) errors in the time a measurement is made (with absolute and drift components); and 3) biological variation in the behaviour of cells or organisms in the assay. Inaccuracy in measurements, leading to noise in the data available for analysis, can, in theory, be addressed by improvement of techniques and by replication. In practice, however, improving measurement quality or increasing replication is often not possible because it can involve slower sampling or result in the inclusion of more biological variation (e.g. through adding parallel cultures, or repeating experiments on different days). Therefore, it is critical to develop analytical approaches which allow robust identification of interactions in biochemical networks from data with a substantial, but poorly-defined, noise component. Such approaches are also valuable in reverse, i.e. in suggesting how experimental sampling strategies can be improved to provide optimal data in terms of both number and accuracy of data points.

Given the ubiquity of measurement noise in biological data, there is clearly a need for advanced estimation algorithms which can explicitly, and in some sense optimally, take such noise into account when producing estimates of the network interactions. In this paper, we consider two such extensions of the classical Least Squares (LS) algorithm, namely the Total Least Squares (TLS) [7,8], and the Constrained Total Least Squares (CTLS) [9,10] algorithm. The CTLS algorithm, in particular, is shown to be ideally suited to the problem of accurately and reliably identifying functional interactions between network components from noisy data. While both of these algorithms are now routinely used in advanced signal and image processing applications, we believe that this is the first time that their usefulness in Systems Biology has been highlighted.

## Results and Discussion

In this section, the performance of the three algorithms described above is tested on an *in silico *four-gene network example, on a high fidelity *in silico *p53 and mdm2 interaction model and on an example of interleukin (IL)-6 and IL- 12b interactions with activating transcription factor 3 (ATF3) and Rel (a component of NF-*κ*B) based on *in vivo *data.

All computations were performed on a 3.06 GHz Pentium IV machine with 1.00 GB of RAM using Windows XP Professional, MATLAB 7.2, and the MATLAB Optimisation Toolbox Version 3.0.4.

### A Four-Gene Network Model

**Figure 1:**
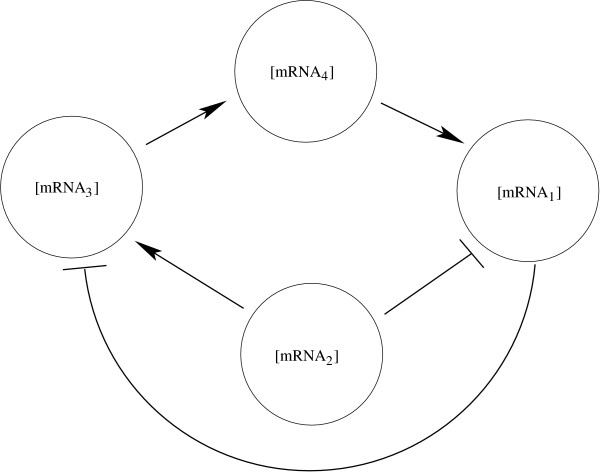
**The four-gene network interactions**. The arrows indicate activating regulatory relationships and the bars indicate inhibiting regulatory relationships. Each messenger RNA is functionally inhibited and/or activated by the expression of other mRNA's. The expression rates are described by the Hill-type equations and given by (1).

A four-gene network example is presented in the supplementary material of [2]. This network was used as a test-bed to evaluate the performance of network identification approaches in both [1] and [2]. The differential equations for the gene network are given by

x˙1(t)=V1s1+A14(x4(t)/K14a)n14[1+(x4(t)/K14a)n14][1+(x2(t)/K12i)n12]−V1dx1(t)k1d+x1(t),     (1a)
 MathType@MTEF@5@5@+=feaafiart1ev1aaatCvAUfKttLearuWrP9MDH5MBPbIqV92AaeXatLxBI9gBaebbnrfifHhDYfgasaacH8akY=wiFfYdH8Gipec8Eeeu0xXdbba9frFj0=OqFfea0dXdd9vqai=hGuQ8kuc9pgc9s8qqaq=dirpe0xb9q8qiLsFr0=vr0=vr0dc8meaabaqaciaacaGaaeqabaqabeGadaaakeaacuWG4baEgaGaamaaBaaaleaacqaIXaqmaeqaaOGaeiikaGIaemiDaqNaeiykaKIaeyypa0JaemOvay1aa0baaSqaaiabigdaXaqaaiabdohaZbaakmaalaaabaGaeGymaeJaey4kaSIaemyqae0aaSbaaSqaaiabigdaXiabisda0aqabaGccqGGOaakcqWG4baEdaWgaaWcbaGaeGinaqdabeaakiabcIcaOiabdsha0jabcMcaPiabc+caViabdUealnaaBaaaleaacqaIXaqmcqaI0aancqWGHbqyaeqaaOGaeiykaKYaaWbaaSqabeaacqWGUbGBdaWgaaadbaGaeGymaeJaeGinaqdabeaaaaaakeaacqGGBbWwcqaIXaqmcqGHRaWkcqGGOaakcqWG4baEdaWgaaWcbaGaeGinaqdabeaakiabcIcaOiabdsha0jabcMcaPiabc+caViabdUealnaaBaaaleaacqaIXaqmcqaI0aancqWGHbqyaeqaaOGaeiykaKYaaWbaaSqabeaacqWGUbGBdaWgaaadbaGaeGymaeJaeGinaqdabeaaaaGccqGGDbqxcqGGBbWwcqaIXaqmcqGHRaWkcqGGOaakcqWG4baEdaWgaaWcbaGaeGOmaidabeaakiabcIcaOiabdsha0jabcMcaPiabc+caViabdUealnaaBaaaleaacqaIXaqmcqaIYaGmcqWGPbqAaeqaaOGaeiykaKYaaWbaaSqabeaacqWGUbGBdaWgaaadbaGaeGymaeJaeGOmaidabeaaaaGccqGGDbqxaaGaeyOeI0IaemOvay1aaSbaaSqaaiabigdaXiabdsgaKbqabaGcdaWcaaqaaiabdIha4naaBaaaleaacqaIXaqmaeqaaOGaeiikaGIaemiDaqNaeiykaKcabaGaem4AaS2aaSbaaSqaaiabigdaXiabdsgaKbqabaGccqGHRaWkcqWG4baEdaWgaaWcbaGaeGymaedabeaakiabcIcaOiabdsha0jabcMcaPaaacqGGSaalcaWLjaGaaCzcamaabmaabaGaeGymaeJaeeyyaegacaGLOaGaayzkaaaaaa@9183@

x˙2(t)=V2s1+A24(x4(t)/K24a)n24[1+(x4(t)/K24a)n24]−V2dx2(t)k2d+x2(t),     (1b)
 MathType@MTEF@5@5@+=feaafiart1ev1aaatCvAUfKttLearuWrP9MDH5MBPbIqV92AaeXatLxBI9gBaebbnrfifHhDYfgasaacH8akY=wiFfYdH8Gipec8Eeeu0xXdbba9frFj0=OqFfea0dXdd9vqai=hGuQ8kuc9pgc9s8qqaq=dirpe0xb9q8qiLsFr0=vr0=vr0dc8meaabaqaciaacaGaaeqabaqabeGadaaakeaacuWG4baEgaGaamaaBaaaleaacqaIYaGmaeqaaOGaeiikaGIaemiDaqNaeiykaKIaeyypa0JaemOvay1aa0baaSqaaiabikdaYaqaaiabdohaZbaakmaalaaabaGaeGymaeJaey4kaSIaemyqae0aaSbaaSqaaiabikdaYiabisda0aqabaGccqGGOaakcqWG4baEdaWgaaWcbaGaeGinaqdabeaakiabcIcaOiabdsha0jabcMcaPiabc+caViabdUealnaaBaaaleaacqaIYaGmcqaI0aancqWGHbqyaeqaaOGaeiykaKYaaWbaaSqabeaacqWGUbGBdaWgaaadbaGaeGOmaiJaeGinaqdabeaaaaaakeaacqGGBbWwcqaIXaqmcqGHRaWkcqGGOaakcqWG4baEdaWgaaWcbaGaeGinaqdabeaakiabcIcaOiabdsha0jabcMcaPiabc+caViabdUealnaaBaaaleaacqaIYaGmcqaI0aancqWGHbqyaeqaaOGaeiykaKYaaWbaaSqabeaacqWGUbGBdaWgaaadbaGaeGOmaiJaeGinaqdabeaaaaGccqGGDbqxaaGaeyOeI0IaemOvay1aaSbaaSqaaiabikdaYiabdsgaKbqabaGcdaWcaaqaaiabdIha4naaBaaaleaacqaIYaGmaeqaaOGaeiikaGIaemiDaqNaeiykaKcabaGaem4AaS2aaSbaaSqaaiabikdaYiabdsgaKbqabaGccqGHRaWkcqWG4baEdaWgaaWcbaGaeGOmaidabeaakiabcIcaOiabdsha0jabcMcaPaaacqGGSaalcaWLjaGaaCzcamaabmaabaGaeGymaeJaeeOyaigacaGLOaGaayzkaaaaaa@7CB0@

x˙3(t)=V3s1+A32(x2(t)/K32a)n32[1+(x2(t)/K32a)n32][1+(x1(t)/K31i)n31]−V1dx3(t)k3d+x3(t),     (1c)
 MathType@MTEF@5@5@+=feaafiart1ev1aaatCvAUfKttLearuWrP9MDH5MBPbIqV92AaeXatLxBI9gBaebbnrfifHhDYfgasaacH8akY=wiFfYdH8Gipec8Eeeu0xXdbba9frFj0=OqFfea0dXdd9vqai=hGuQ8kuc9pgc9s8qqaq=dirpe0xb9q8qiLsFr0=vr0=vr0dc8meaabaqaciaacaGaaeqabaqabeGadaaakeaacuWG4baEgaGaamaaBaaaleaacqaIZaWmaeqaaOGaeiikaGIaemiDaqNaeiykaKIaeyypa0JaemOvay1aa0baaSqaaiabiodaZaqaaiabdohaZbaakmaalaaabaGaeGymaeJaey4kaSIaemyqae0aaSbaaSqaaiabiodaZiabikdaYaqabaGccqGGOaakcqWG4baEdaWgaaWcbaGaeGOmaidabeaakiabcIcaOiabdsha0jabcMcaPiabc+caViabdUealnaaBaaaleaacqaIZaWmcqaIYaGmcqWGHbqyaeqaaOGaeiykaKYaaWbaaSqabeaacqWGUbGBdaWgaaadbaGaeG4mamJaeGOmaidabeaaaaaakeaacqGGBbWwcqaIXaqmcqGHRaWkcqGGOaakcqWG4baEdaWgaaWcbaGaeGOmaidabeaakiabcIcaOiabdsha0jabcMcaPiabc+caViabdUealnaaBaaaleaacqaIZaWmcqaIYaGmcqWGHbqyaeqaaOGaeiykaKYaaWbaaSqabeaacqWGUbGBdaWgaaadbaGaeG4mamJaeGOmaidabeaaaaGccqGGDbqxcqGGBbWwcqaIXaqmcqGHRaWkcqGGOaakcqWG4baEdaWgaaWcbaGaeGymaedabeaakiabcIcaOiabdsha0jabcMcaPiabc+caViabdUealnaaBaaaleaacqaIZaWmcqaIXaqmcqWGPbqAaeqaaOGaeiykaKYaaWbaaSqabeaacqWGUbGBdaWgaaadbaGaeG4mamJaeGymaedabeaaaaGccqGGDbqxaaGaeyOeI0IaemOvay1aaSbaaSqaaiabigdaXiabdsgaKbqabaGcdaWcaaqaaiabdIha4naaBaaaleaacqaIZaWmaeqaaOGaeiikaGIaemiDaqNaeiykaKcabaGaem4AaS2aaSbaaSqaaiabiodaZiabdsgaKbqabaGccqGHRaWkcqWG4baEdaWgaaWcbaGaeG4mamdabeaakiabcIcaOiabdsha0jabcMcaPaaacqGGSaalcaWLjaGaaCzcamaabmaabaGaeGymaeJaee4yamgacaGLOaGaayzkaaaaaa@9195@

x˙4(t)=V4s1+A43(x3(t)/K43a)n43[1+(x3(t)/K43a)n43]−V4dx4(t)k4d+x4(t)     (1d)
 MathType@MTEF@5@5@+=feaafiart1ev1aaatCvAUfKttLearuWrP9MDH5MBPbIqV92AaeXatLxBI9gBaebbnrfifHhDYfgasaacH8akY=wiFfYdH8Gipec8Eeeu0xXdbba9frFj0=OqFfea0dXdd9vqai=hGuQ8kuc9pgc9s8qqaq=dirpe0xb9q8qiLsFr0=vr0=vr0dc8meaabaqaciaacaGaaeqabaqabeGadaaakeaacuWG4baEgaGaamaaBaaaleaacqaI0aanaeqaaOGaeiikaGIaemiDaqNaeiykaKIaeyypa0JaemOvay1aa0baaSqaaiabisda0aqaaiabdohaZbaakmaalaaabaGaeGymaeJaey4kaSIaemyqae0aaSbaaSqaaiabisda0iabiodaZaqabaGccqGGOaakcqWG4baEdaWgaaWcbaGaeG4mamdabeaakiabcIcaOiabdsha0jabcMcaPiabc+caViabdUealnaaBaaaleaacqaI0aancqaIZaWmcqWGHbqyaeqaaOGaeiykaKYaaWbaaSqabeaacqWGUbGBdaWgaaadbaGaeGinaqJaeG4mamdabeaaaaaakeaacqGGBbWwcqaIXaqmcqGHRaWkcqGGOaakcqWG4baEdaWgaaWcbaGaeG4mamdabeaakiabcIcaOiabdsha0jabcMcaPiabc+caViabdUealnaaBaaaleaacqaI0aancqaIZaWmcqWGHbqyaeqaaOGaeiykaKYaaWbaaSqabeaacqWGUbGBdaWgaaadbaGaeGinaqJaeG4mamdabeaaaaGccqGGDbqxaaGaeyOeI0IaemOvay1aaSbaaSqaaiabisda0iabdsgaKbqabaGcdaWcaaqaaiabdIha4naaBaaaleaacqaI0aanaeqaaOGaeiikaGIaemiDaqNaeiykaKcabaGaem4AaS2aaSbaaSqaaiabisda0iabdsgaKbqabaGccqGHRaWkcqWG4baEdaWgaaWcbaGaeGinaqdabeaakiabcIcaOiabdsha0jabcMcaPaaacaWLjaGaaCzcamaabmaabaGaeGymaeJaeeizaqgacaGLOaGaayzkaaaaaa@7BF2@

where *x*_*i*_(*t*) is the concentration of mRNA_*i*_, for *i *= 1, 2, 3, 4, the first term and the second term on the right hand side of the equations represent the rate of transcription and the rate of degradation of each mRNA, respectively, and each maximal enzyme rate is given by V1s
 MathType@MTEF@5@5@+=feaafiart1ev1aaatCvAUfKttLearuWrP9MDH5MBPbIqV92AaeXatLxBI9gBaebbnrfifHhDYfgasaacH8akY=wiFfYdH8Gipec8Eeeu0xXdbba9frFj0=OqFfea0dXdd9vqai=hGuQ8kuc9pgc9s8qqaq=dirpe0xb9q8qiLsFr0=vr0=vr0dc8meaabaqaciaacaGaaeqabaqabeGadaaakeaacqWGwbGvdaqhaaWcbaGaeGymaedabaGaem4Camhaaaaa@306D@ = 5, V2s
 MathType@MTEF@5@5@+=feaafiart1ev1aaatCvAUfKttLearuWrP9MDH5MBPbIqV92AaeXatLxBI9gBaebbnrfifHhDYfgasaacH8akY=wiFfYdH8Gipec8Eeeu0xXdbba9frFj0=OqFfea0dXdd9vqai=hGuQ8kuc9pgc9s8qqaq=dirpe0xb9q8qiLsFr0=vr0=vr0dc8meaabaqaciaacaGaaeqabaqabeGadaaakeaacqWGwbGvdaqhaaWcbaGaeGOmaidabaGaem4Camhaaaaa@306F@ = 3.5, V3s
 MathType@MTEF@5@5@+=feaafiart1ev1aaatCvAUfKttLearuWrP9MDH5MBPbIqV92AaeXatLxBI9gBaebbnrfifHhDYfgasaacH8akY=wiFfYdH8Gipec8Eeeu0xXdbba9frFj0=OqFfea0dXdd9vqai=hGuQ8kuc9pgc9s8qqaq=dirpe0xb9q8qiLsFr0=vr0=vr0dc8meaabaqaciaacaGaaeqabaqabeGadaaakeaacqWGwbGvdaqhaaWcbaGaeG4mamdabaGaem4Camhaaaaa@3071@ = 3, V4s
 MathType@MTEF@5@5@+=feaafiart1ev1aaatCvAUfKttLearuWrP9MDH5MBPbIqV92AaeXatLxBI9gBaebbnrfifHhDYfgasaacH8akY=wiFfYdH8Gipec8Eeeu0xXdbba9frFj0=OqFfea0dXdd9vqai=hGuQ8kuc9pgc9s8qqaq=dirpe0xb9q8qiLsFr0=vr0=vr0dc8meaabaqaciaacaGaaeqabaqabeGadaaakeaacqWGwbGvdaqhaaWcbaGaeGinaqdabaGaem4Camhaaaaa@3073@ = 4, V1d
 MathType@MTEF@5@5@+=feaafiart1ev1aaatCvAUfKttLearuWrP9MDH5MBPbIqV92AaeXatLxBI9gBaebbnrfifHhDYfgasaacH8akY=wiFfYdH8Gipec8Eeeu0xXdbba9frFj0=OqFfea0dXdd9vqai=hGuQ8kuc9pgc9s8qqaq=dirpe0xb9q8qiLsFr0=vr0=vr0dc8meaabaqaciaacaGaaeqabaqabeGadaaakeaacqWGwbGvdaqhaaWcbaGaeGymaedabaGaemizaqgaaaaa@304F@ = 200, V2d
 MathType@MTEF@5@5@+=feaafiart1ev1aaatCvAUfKttLearuWrP9MDH5MBPbIqV92AaeXatLxBI9gBaebbnrfifHhDYfgasaacH8akY=wiFfYdH8Gipec8Eeeu0xXdbba9frFj0=OqFfea0dXdd9vqai=hGuQ8kuc9pgc9s8qqaq=dirpe0xb9q8qiLsFr0=vr0=vr0dc8meaabaqaciaacaGaaeqabaqabeGadaaakeaacqWGwbGvdaqhaaWcbaGaeGOmaidabaGaemizaqgaaaaa@3051@ = 500, V3d
 MathType@MTEF@5@5@+=feaafiart1ev1aaatCvAUfKttLearuWrP9MDH5MBPbIqV92AaeXatLxBI9gBaebbnrfifHhDYfgasaacH8akY=wiFfYdH8Gipec8Eeeu0xXdbba9frFj0=OqFfea0dXdd9vqai=hGuQ8kuc9pgc9s8qqaq=dirpe0xb9q8qiLsFr0=vr0=vr0dc8meaabaqaciaacaGaaeqabaqabeGadaaakeaacqWGwbGvdaqhaaWcbaGaeG4mamdabaGaemizaqgaaaaa@3053@ = 150, V4d
 MathType@MTEF@5@5@+=feaafiart1ev1aaatCvAUfKttLearuWrP9MDH5MBPbIqV92AaeXatLxBI9gBaebbnrfifHhDYfgasaacH8akY=wiFfYdH8Gipec8Eeeu0xXdbba9frFj0=OqFfea0dXdd9vqai=hGuQ8kuc9pgc9s8qqaq=dirpe0xb9q8qiLsFr0=vr0=vr0dc8meaabaqaciaacaGaaeqabaqabeGadaaakeaacqWGwbGvdaqhaaWcbaGaeGinaqdabaGaemizaqgaaaaa@3055@ = 500, with units of nM · h^-1^. The Michaelis constants are given by *K*_14*a *_= 1.6, *K*_24*a *_= 1.6, *K*_32*a *_= 1.5, *K*_43*a *_= 0.15, *K*_12*i *_= 0.5, *K*_31*i *_= 0.7, *K*_1*d *_= 30, *K*_2*d *_= 60, *K*_3*d *_= 10, *K*_4*d *_= 50, in units of nM, and *A*_14 _= 4, *A*_24 _= 4, *A*_32 _= 5, *A*_43 _= 2, *n*_12 _= 1, *n*_14 _= 2, *n*_24 _= 2, *n*_31 _= 1, *n*_32 _= 2, *n*_43 _= 2. In the model, gene interactions result in nonlinear dependencies of transcription rates on other mRNA concentrations, which act as communicating intermediaries. The corresponding gene network for this example is shown in Figure 1.

**Figure 2:**
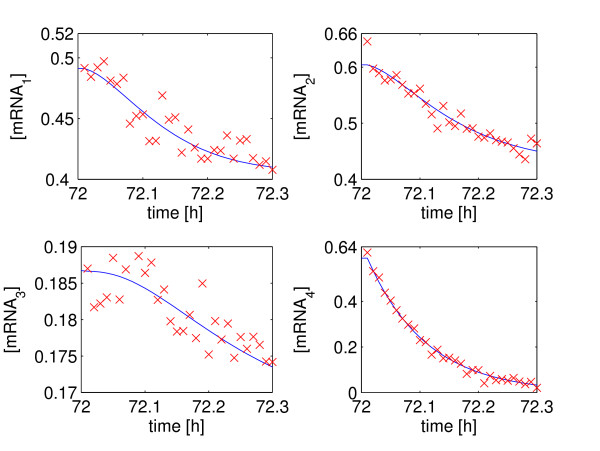
**The four-gene network measurements corrupted by white noise**. Measurements of 30 noisy data points for each mRNA concentration are shown. The solid line represents the true simulated value and the crosses denote the measurements corrupted by white noise. The measurements are taken starting at 72.01 h, 0.01 h after the perturbation is applied.

For this example, the level of perturbation for Vis
 MathType@MTEF@5@5@+=feaafiart1ev1aaatCvAUfKttLearuWrP9MDH5MBPbIqV92AaeXatLxBI9gBaebbnrfifHhDYfgasaacH8akY=wiFfYdH8Gipec8Eeeu0xXdbba9frFj0=OqFfea0dXdd9vqai=hGuQ8kuc9pgc9s8qqaq=dirpe0xb9q8qiLsFr0=vr0=vr0dc8meaabaqaciaacaGaaeqabaqabeGadaaakeaacqWGwbGvdaqhaaWcbaGaemyAaKgabaGaem4Camhaaaaa@30D8@ for *i *= 1, 2, 3, 4 from the nominal values is 100% and the measurement noise is assumed to be zero-mean white gaussian with variance equal to the square of the equilibrium times 0.02, where the equilibrium states are given by x1eq
 MathType@MTEF@5@5@+=feaafiart1ev1aaatCvAUfKttLearuWrP9MDH5MBPbIqV92AaeXatLxBI9gBaebbnrfifHhDYfgasaacH8akY=wiFfYdH8Gipec8Eeeu0xXdbba9frFj0=OqFfea0dXdd9vqai=hGuQ8kuc9pgc9s8qqaq=dirpe0xb9q8qiLsFr0=vr0=vr0dc8meaabaqaciaacaGaaeqabaqabeGadaaakeaacqWG4baEdaqhaaWcbaGaeGymaedabaGaeeyzauMaeeyCaehaaaaa@31FC@ = 0.4920, x2eq
 MathType@MTEF@5@5@+=feaafiart1ev1aaatCvAUfKttLearuWrP9MDH5MBPbIqV92AaeXatLxBI9gBaebbnrfifHhDYfgasaacH8akY=wiFfYdH8Gipec8Eeeu0xXdbba9frFj0=OqFfea0dXdd9vqai=hGuQ8kuc9pgc9s8qqaq=dirpe0xb9q8qiLsFr0=vr0=vr0dc8meaabaqaciaacaGaaeqabaqabeGadaaakeaacqWG4baEdaqhaaWcbaGaeGOmaidabaGaeeyzauMaeeyCaehaaaaa@31FE@ = 0.6052, x3eq
 MathType@MTEF@5@5@+=feaafiart1ev1aaatCvAUfKttLearuWrP9MDH5MBPbIqV92AaeXatLxBI9gBaebbnrfifHhDYfgasaacH8akY=wiFfYdH8Gipec8Eeeu0xXdbba9frFj0=OqFfea0dXdd9vqai=hGuQ8kuc9pgc9s8qqaq=dirpe0xb9q8qiLsFr0=vr0=vr0dc8meaabaqaciaacaGaaeqabaqabeGadaaakeaacqWG4baEdaqhaaWcbaGaeG4mamdabaGaeeyzauMaeeyCaehaaaaa@3200@ = 0.1866, and x4eq
 MathType@MTEF@5@5@+=feaafiart1ev1aaatCvAUfKttLearuWrP9MDH5MBPbIqV92AaeXatLxBI9gBaebbnrfifHhDYfgasaacH8akY=wiFfYdH8Gipec8Eeeu0xXdbba9frFj0=OqFfea0dXdd9vqai=hGuQ8kuc9pgc9s8qqaq=dirpe0xb9q8qiLsFr0=vr0=vr0dc8meaabaqaciaacaGaaeqabaqabeGadaaakeaacqWG4baEdaqhaaWcbaGaeGinaqdabaGaeeyzauMaeeyCaehaaaaa@3202@ = 0.6514. The number of experiments is four. In each experiment Vis
 MathType@MTEF@5@5@+=feaafiart1ev1aaatCvAUfKttLearuWrP9MDH5MBPbIqV92AaeXatLxBI9gBaebbnrfifHhDYfgasaacH8akY=wiFfYdH8Gipec8Eeeu0xXdbba9frFj0=OqFfea0dXdd9vqai=hGuQ8kuc9pgc9s8qqaq=dirpe0xb9q8qiLsFr0=vr0=vr0dc8meaabaqaciaacaGaaeqabaqabeGadaaakeaacqWGwbGvdaqhaaWcbaGaemyAaKgabaGaem4Camhaaaaa@30D8@ is perturbed in the negative direction, i.e. inhibited, and the data sampling time is 0.01 h (36s). The true (simulated) values of *x*_*i*_(*t*) together with the noisy measurements for this example are shown in Figure 2.

The three different least squares algorithms are tested for different numbers of data points per experiment, i.e., 3, 6, 9, 12, 21, 30, 60, and the quality of the Jacobian estimations was evaluated according to a number of different definitions of estimation error, which are discussed in the Methods section. The results generated from 1000 Monte-Carlo simulations are given in Table 1.

**Table 1:** The four-gene network example: white noise

Samplings per Experiment	Algorithms	*ε*_*M*_		*ε*_*S*_		*ε*_*F*_	
		Mean	STD	Mean	STD	Mean	STD
3	LS	94.36	36.54	0.95	0.20	368.06	123.08
	TLS	94.36	36.54	0.95	0.20	368.06	123.08
	CTLS	94.36	36.54	0.95	0.20	368.06	123.08

6	LS	16.35	5.11	0.59	0.14	71.10	17.84
	TLS	196.04	2239.78	0.74	0.20	1778.75	24252.19
	CTLS	14.96	5.63	0.63	0.16	64.29	21.34

9	LS	7.87	2.42	0.46	0.09	35.73	9.03
	TLS	11.96	9.68	0.54	0.13	67.47	118.27
	CTLS	6.61	2.74	0.47	0.10	31.57	12.05

12	LS	5.19	1.64	0.40	0.06	24.98	6.47
	TLS	6.20	2.34	0.45	0.09	32.42	15.33
	CTLS	3.79	1.48	0.40	0.06	19.59	6.74

21	LS	3.74	1.06	0.38	0.02	18.12	4.39
	TLS	3.71	1.36	0.40	0.05	20.40	8.51
	CTLS	2.20	0.68	0.38	0.02	11.29	2.93

30	LS	3.70	0.87	0.41	0.06	17.21	3.62
	TLS	3.45	1.20	0.44	0.07	18.75	7.30
	CTLS	2.31	0.56	0.49	0.03	10.10	1.96

60	LS	3.75	0.66	0.50	0.01	17.05	2.59
	TLS	3.59	1.05	0.52	0.05	16.25	4.74
	CTLS	2.51	0.52	0.50	0.01	10.76	1.45

The table shows the error comparisons in terms of the mean and the standard deviation (STD) for different numbers of data points for each method based on 1000 Monte-Carlo Simulations. *ε*_*M *_is the sum of two terms, i.e (l/*N*_1_) Σ |*α*_*ij*_| and (l/*N*_2_) Σ |*β*_*ij*_| where *α*_*ij *_and *β*_*ij *_are the relative magnitude errors in the non-zero and zero elements of the true Jacobian, respectively, and *N*_1 _and *N*_2 _are the number of non-zero and zero elements in the true Jacobian, respectively. *ε*_*S *_is given by (1/*n*^2^) Σ |sign (f^
 MathType@MTEF@5@5@+=feaafiart1ev1aaatCvAUfKttLearuWrP9MDH5MBPbIqV92AaeXatLxBI9gBaebbnrfifHhDYfgasaacH8akY=wiFfYdH8Gipec8Eeeu0xXdbba9frFj0=OqFfea0dXdd9vqai=hGuQ8kuc9pgc9s8qqaq=dirpe0xb9q8qiLsFr0=vr0=vr0dc8meaabaqaciaacaGaaeqabaqabeGadaaakeaacuWGMbGzgaqcaaaa@2E11@_*ij*_) - sign (*f*_*ij*_)|, i.e. the average sign differences, where f^
 MathType@MTEF@5@5@+=feaafiart1ev1aaatCvAUfKttLearuWrP9MDH5MBPbIqV92AaeXatLxBI9gBaebbnrfifHhDYfgasaacH8akY=wiFfYdH8Gipec8Eeeu0xXdbba9frFj0=OqFfea0dXdd9vqai=hGuQ8kuc9pgc9s8qqaq=dirpe0xb9q8qiLsFr0=vr0=vr0dc8meaabaqaciaacaGaaeqabaqabeGadaaakeaacuWGMbGzgaqcaaaa@2E11@_*ij *_and *f*_*ij *_are the (*i*-th row, *j*-th column) elements of the estimated and the true Jacobian, respectively. *ε*_*F *_is the Frobenius norm of the difference between the estimated and the true Jacobian, i.e. ||F^
 MathType@MTEF@5@5@+=feaafiart1ev1aaatCvAUfKttLearuWrP9MDH5MBPbIqV92AaeXatLxBI9gBaebbnrfifHhDYfgasaacH8akY=wiFfYdH8Gipec8Eeeu0xXdbba9frFj0=OqFfea0dXdd9vqai=hGuQ8kuc9pgc9s8qqaq=dirpe0xb9q8qiLsFr0=vr0=vr0dc8meaabaqaciaacaGaaeqabaqabeGadaaakeaacuWGgbGrgaqcaaaa@2DD1@ - *F*||_*F*_.

Note that the estimation errors of the TLS for the cases of very few data points, i.e. 6, 9, and 12 are larger than the errors from the standard least squares algorithm. This is because, as discussed later in the Methods section, the TLS algorithm requires a minimum number of data points to work properly. For the case of only 3 data points, all three algorithms provide the same result, since in this case the set of equations to be solved is not over-determined, i.e. there is a single unique solution. Excluding this case, the CTLS reduces the mean of the relative magnitude error for each element of the Jacobian, i.e. *ε*_*M*_, by an average of 27% compared with the standard least squares technique, over all the different cases considered. This improvement rises to 37% when the four cases with the fewest data points are removed. The variance of the error is reduced by an average of 25.6%, excluding the first three cases. For the sign estimation error, *ε*_*S*_, all three methods give a similar level of performance – the reason for this is easy to see, however, by considering the true Jacobian of the network:

F=[−6.45−2.9202.540−8.1703.93−2.312.80−14.4600010.22−9.74].     (2)
 MathType@MTEF@5@5@+=feaafiart1ev1aaatCvAUfKttLearuWrP9MDH5MBPbIqV92AaeXatLxBI9gBaebbnrfifHhDYfgasaacH8akY=wiFfYdH8Gipec8Eeeu0xXdbba9frFj0=OqFfea0dXdd9vqai=hGuQ8kuc9pgc9s8qqaq=dirpe0xb9q8qiLsFr0=vr0=vr0dc8meaabaqaciaacaGaaeqabaqabeGadaaakeaacqWGgbGrcqGH9aqpdaWadaqaauaabeqaeqaaaaaabaGaeyOeI0IaeGOnayJaeiOla4IaeGinaqJaeGynaudabaGaeyOeI0IaeGOmaiJaeiOla4IaeGyoaKJaeGOmaidabaGaeGimaadabaGaeGOmaiJaeiOla4IaeGynauJaeGinaqdabaGaeGimaadabaGaeyOeI0IaeGioaGJaeiOla4IaeGymaeJaeG4naCdabaGaeGimaadabaGaeG4mamJaeiOla4IaeGyoaKJaeG4mamdabaGaeyOeI0IaeGOmaiJaeiOla4IaeG4mamJaeGymaedabaGaeGOmaiJaeiOla4IaeGioaGJaeGimaadabaGaeyOeI0IaeGymaeJaeGinaqJaeiOla4IaeGinaqJaeGOnaydabaGaeGimaadabaGaeGimaadabaGaeGimaadabaGaeGymaeJaeGimaaJaeiOla4IaeGOmaiJaeGOmaidabaGaeyOeI0IaeGyoaKJaeiOla4IaeG4naCJaeGinaqdaaaGaay5waiaaw2faaiabc6caUiaaxMaacaWLjaWaaeWaaeaacqaIYaGmaiaawIcacaGLPaaaaaa@6842@

Clearly, the Jacobian contains no terms which are very close to zero and therefore the signs of the estimates for each term will be very similar for all three methods. The CTLS almost always gives the best performance in the root mean square sense. A common feature of the results presented in Table 1 is that the accuracy of the estimate improves with increasing numbers of data points. However, beyond a certain critical number of data points, there is no further improvement in the quality of the estimate using any algorithm. The fact that for certain error measures the estimate dis-improves slightly for a large number of data points is due to the biased nature of the least squares solution. It is well known that when *A *and *b *in *Ax *= *b *are statistically independent, the least square solution has no bias error. However, when they are not independent, which is the case here, the solution has a bias in general. Moreover, the level of bias is generally a nonlinear function of the number of measurements and hence the bias error may not decrease monotonically [11]. From Table 1, it is clear that using too few data points can generate huge errors in the inferred network. However, since the error does not decrease monotonically with larger number of data points, it may be better to increase the accuracy of the data while obtaining fewer data points rather than sacrificing accuracy to obtain many more data points. In this specific case, the optimal number of data points seems to be between 21 and 60. Finally, to evaluate the effect of drift noise, which is a common form of noise in biochemical measurements, each algorithm was again evaluated using Monte-Carlo simulations. In this case, the measurements are given by [12]

Δx˜
 MathType@MTEF@5@5@+=feaafiart1ev1aaatCvAUfKttLearuWrP9MDH5MBPbIqV92AaeXatLxBI9gBaebbnrfifHhDYfgasaacH8akY=wiFfYdH8Gipec8Eeeu0xXdbba9frFj0=OqFfea0dXdd9vqai=hGuQ8kuc9pgc9s8qqaq=dirpe0xb9q8qiLsFr0=vr0=vr0dc8meaabaqaciaacaGaaeqabaqabeGadaaakeaacuWG4baEgaacaaaa@2E34@ = Δ*x*_*k *_+ *v*_*k *_+ *b*_*k *_    (3)

for *k *= 1, 2, ..., *L *where *v*_*k *_is white noise and *b*_*k *_is the drift noise. The drift noise, which is also called Brownian motion or random walk, is modelled as follows:

*b*_*k*+1 _= *b*_*k *_+ *w*_*k*_Δ*T *    (4)

for *k *= 1, 2, ..., *L *- 1 where *b*_1 _equals to 0 and *w*_*k *_is white noise. The dimensions of *b*_*k *_and *w*_*k *_are *n *× 1 and the variance of the *i*-th element of *w*_*k *_is (*x_i_*^eq^*γ*)^2 ^for *i *= 1, 2, ..., *n*. For this example, *n *is equal to 4. Monte-Carlo simulation results for various levels of drift noise are shown in Tables 2 and 3, for 12 and 21 data points per experiment, respectively. Again, for each case, and for all different levels of drift noise considered, the CTLS algorithm generally yields significant reductions in the Jacobian estimation error. By comparing Tables 2 and 3, it can be seen that the errors with 21 data points are smaller than the ones with 12 data points. However, if the number of data points is increased by too much, adverse effects will occur, as the bias error is stronger as time increases and hence the later data are effected more strongly by bias noise. Hence, in the presence of bias noise it is also important to try to choose the optimal number of data points so that the noise does not increase the estimation error too much.

**Table 2:** The four-gene network example: 12 data points, white noise and drift noise

Strength of drift noise (*γ*)	Algorithms	*ε*_*M*_		*ε*_*S*_		*ε*_*F*_	
		Mean	STD	Mean	STD	Mean	STD
2.0	LS	9.18	3.63	0.47	0.10	41.38	14.19
	TLS	29.25	178.08	0.57	0.15	237.51	2995.95
	CTLS	8.37	3.95	0.51	0.12	40.28	20.33

1.0	LS	6.31	2.07	0.42	0.07	29.24	8.25
	TLS	8.21	5.02	0.48	0.11	41.76	28.25
	CTLS	5.01	2.00	0.43	0.09	24.67	9.62

0.1	LS	5.14	1.59	0.40	0.06	25.02	6.61
	TLS	6.21	2.38	0.45	0.09	32.87	15.91
	CTLS	3.79	1.40	0.40	0.05	19.71	6.89

0.05	LS	5.18	1.66	0.40	0.06	25.16	6.57
	TLS	6.20	2.39	0.45	0.09	32.29	15.30
	CTLS	3.79	1.46	0.40	0.06	19.56	6.80

The table shows the error comparisons in terms of the mean and the standard deviation (STD) for different strengths of drift noise for each method based on 1000 Monte-Carlo simulations. The number of measurements per experiment is fixed at 12. All conditions are the same as in Table 1 with only the drift noise being added. *ε*_*M *_is the sum of two tems, i.e (1/*N*_1_) Σ |*α*_*i j*_| and (1/*N*_2_) Σ |*β*_*i j*_|, where *β*_*i j *_and *β*_*i j *_are the relative magnitude errors in the non-zero and zero elements of the true Jacobian, respectively, and *N*_1 _and *N*_2 _are the number of non-zero and zero elements in the true Jacobian, respectively. *ε*_*S *_is given by (1/*n*^2^) Σ |sign (f^
 MathType@MTEF@5@5@+=feaafiart1ev1aaatCvAUfKttLearuWrP9MDH5MBPbIqV92AaeXatLxBI9gBaebbnrfifHhDYfgasaacH8akY=wiFfYdH8Gipec8Eeeu0xXdbba9frFj0=OqFfea0dXdd9vqai=hGuQ8kuc9pgc9s8qqaq=dirpe0xb9q8qiLsFr0=vr0=vr0dc8meaabaqaciaacaGaaeqabaqabeGadaaakeaacuWGMbGzgaqcaaaa@2E11@_*i j*_) - sign(*f*_*i j*_)|, i.e. the average sign differences, where f^
 MathType@MTEF@5@5@+=feaafiart1ev1aaatCvAUfKttLearuWrP9MDH5MBPbIqV92AaeXatLxBI9gBaebbnrfifHhDYfgasaacH8akY=wiFfYdH8Gipec8Eeeu0xXdbba9frFj0=OqFfea0dXdd9vqai=hGuQ8kuc9pgc9s8qqaq=dirpe0xb9q8qiLsFr0=vr0=vr0dc8meaabaqaciaacaGaaeqabaqabeGadaaakeaacuWGMbGzgaqcaaaa@2E11@_*i j *_and *f*_*i j *_are the (*i*-th row, *j*-th column) elements of the estimated and the true Jacobian, respectively. *ε*_*F *_is the Frobenius norm of the difference between the estimated and the true Jacobian, i.e. ||F^
 MathType@MTEF@5@5@+=feaafiart1ev1aaatCvAUfKttLearuWrP9MDH5MBPbIqV92AaeXatLxBI9gBaebbnrfifHhDYfgasaacH8akY=wiFfYdH8Gipec8Eeeu0xXdbba9frFj0=OqFfea0dXdd9vqai=hGuQ8kuc9pgc9s8qqaq=dirpe0xb9q8qiLsFr0=vr0=vr0dc8meaabaqaciaacaGaaeqabaqabeGadaaakeaacuWGgbGrgaqcaaaa@2DD1@ - *F*||_*F*_.

**Table 3:** The four-gene network example: 21 data points, white noise and drift noise

Strength of drift noise (*γ*)	Algorithms	*ε*_*M*_		*ε*_*S*_		*ε*_*F*_	
		Mean	STD	Mean	STD	Mean	STD
2.0	LS	6.32	2.59	0.43	0.08	28.32	10.61
	TLS	19.57	179.54	0.50	0.12	111.66	949.78
	CTLS	5.81	3.10	0.47	0.10	28.62	17.56

1.0	LS	4.46	1.51	0.39	0.05	21.21	6.61
	TLS	4.83	2.15	0.43	0.08	26.42	14.98
	CTLS	3.17	1.24	0.41	0.06	16.08	7.20

0.1	LS	3.68	1.05	0.38	0.02	17.93	4.34
	TLS	3.58	1.27	0.40	0.05	19.90	8.33
	CTLS	2.18	0.68	0.38	0.02	11.16	2.91

0.05	LS	3.68	1.04	0.38	0.02	17.92	4.10
	TLS	3.63	1.35	0.40	0.06	19.88	8.16
	CTLS	2.18	0.69	0.38	0.02	11.14	2.93

The table shows the error comparisons in terms of the mean and the standard deviation (STD) for different strengths of drift noise for each method based on 1000 Monte-Carlo simulations. The number of measurements per experiment is fixed at 21. All conditions are the same as the ones for Table 1 with only the drift noise being added. *ε*_*M *_is the sum of two tems, i.e (1/*N*_1_) Σ |*α*_*i j*_| and (l/*N*_2_) Σ |*β*_*i j*_|, where *β*_*i j *_and *β*_*i j *_are the relative magnitude errors in the non-zero and zero elements of the true Jacobian, respectively, and *N*_1 _and *N*_2 _are the number of non-zero and zero elements in the true Jacobian, respectively. *ε*_*S *_is given by (1/*n*^2^) Σ |sign(f^
 MathType@MTEF@5@5@+=feaafiart1ev1aaatCvAUfKttLearuWrP9MDH5MBPbIqV92AaeXatLxBI9gBaebbnrfifHhDYfgasaacH8akY=wiFfYdH8Gipec8Eeeu0xXdbba9frFj0=OqFfea0dXdd9vqai=hGuQ8kuc9pgc9s8qqaq=dirpe0xb9q8qiLsFr0=vr0=vr0dc8meaabaqaciaacaGaaeqabaqabeGadaaakeaacuWGMbGzgaqcaaaa@2E11@_*i j*_) - sign(*f*_*i j*_)|, i.e. the average sign differences, where f^
 MathType@MTEF@5@5@+=feaafiart1ev1aaatCvAUfKttLearuWrP9MDH5MBPbIqV92AaeXatLxBI9gBaebbnrfifHhDYfgasaacH8akY=wiFfYdH8Gipec8Eeeu0xXdbba9frFj0=OqFfea0dXdd9vqai=hGuQ8kuc9pgc9s8qqaq=dirpe0xb9q8qiLsFr0=vr0=vr0dc8meaabaqaciaacaGaaeqabaqabeGadaaakeaacuWGMbGzgaqcaaaa@2E11@_*i j *_and *f*_*i j *_are the (*i*-th row, *j*-th column) element of the estimated and the true Jacobian, respectively. *ε*_*F *_is the Frobenius norm of the difference between the estimated and the true Jacobian, i.e. ||F^
 MathType@MTEF@5@5@+=feaafiart1ev1aaatCvAUfKttLearuWrP9MDH5MBPbIqV92AaeXatLxBI9gBaebbnrfifHhDYfgasaacH8akY=wiFfYdH8Gipec8Eeeu0xXdbba9frFj0=OqFfea0dXdd9vqai=hGuQ8kuc9pgc9s8qqaq=dirpe0xb9q8qiLsFr0=vr0=vr0dc8meaabaqaciaacaGaaeqabaqabeGadaaakeaacuWGgbGrgaqcaaaa@2DD1@ - *F*||_*F*_.

### p53 and *mdm2 *messenger RNA expression

The negative feedback interactions between the tumor suppressor p53 and the oncogene Mdm2 have been the subject of much attention in the recent Systems Biology literature. p53 protein activates *mdm2 *and the Mdm2 protein in turn negatively regulates p53, forming a negative feedback loop. The nature of the oscillations in p53 have been observed to vary significantly from cell to cell. The period and the amplitude variability seem to stem from low frequency noise in the protein production rate, [13]. A detailed mathematical model for the single-cell response of p53 to ionising radiation, which includes the levels of p53 and transcription of the *mdm2 *gene, the corresponding protein levels of mdm2 and the activation kinetics of the protein p53 and ataxia telangiectasia mutated (ATM), is presented in [14]. The model accurately replicates the experimentally observed phenomenon that the number, but not the frequency or amplitude, of p53 oscillations depends on the radiation dose. The full model includes not only deterministic but also some stochastic dynamics, which represent the stochastic behaviour of the number of double-strand break complexes. To formulate a realistic problem to demonstrate the performance of the constrained total least-squares algorithm, we considered the following scenario: First, the p53 activity and *mdm2 *gene expression levels, as shown in Figure 7A of [14], are the only measurements available. Second, we do not know the activity of ATM and other proteins including p53. Finally, the relation between these two genes is to be estimated by the Jacobian estimation algorithm. The kinetics for the two gene expression levels are given by

d[p53]dt=sp53−δp53[p53],     (5a)
 MathType@MTEF@5@5@+=feaafiart1ev1aaatCvAUfKttLearuWrP9MDH5MBPbIqV92AaeXatLxBI9gBaebbnrfifHhDYfgasaacH8akY=wiFfYdH8Gipec8Eeeu0xXdbba9frFj0=OqFfea0dXdd9vqai=hGuQ8kuc9pgc9s8qqaq=dirpe0xb9q8qiLsFr0=vr0=vr0dc8meaabaqaciaacaGaaeqabaqabeGadaaakeaadaWcaaqaaiabdsgaKjabcUfaBjabbchaWjabiwda1iabiodaZiabc2faDbqaaiabdsgaKjabdsha0baacqGH9aqpcqWGZbWCdaWgaaWcbaGaeeiCaaNaeGynauJaeG4mamdabeaakiabgkHiTGGaciab=r7aKnaaBaaaleaacqqGWbaCcqaI1aqncqaIZaWmaeqaaOGaei4waSLaeeiCaaNaeGynauJaeG4mamJaeiyxa0LaeiilaWIaaCzcaiaaxMaadaqadaqaaiabiwda1iabbggaHbGaayjkaiaawMcaaaaa@4E83@

d[mdm2]dt=smdm2−δmdm2[mdm2]+εmdm2[p53*(t−τ1)]n[p53*(t−τ1)]n+Kn,     (5b)
 MathType@MTEF@5@5@+=feaafiart1ev1aaatCvAUfKttLearuWrP9MDH5MBPbIqV92AaeXatLxBI9gBaebbnrfifHhDYfgasaacH8akY=wiFfYdH8Gipec8Eeeu0xXdbba9frFj0=OqFfea0dXdd9vqai=hGuQ8kuc9pgc9s8qqaq=dirpe0xb9q8qiLsFr0=vr0=vr0dc8meaabaqaciaacaGaaeqabaqabeGadaaakeaadaWcaaqaaiabdsgaKjabcUfaBjabd2gaTjabdsgaKjabd2gaTjabikdaYiabc2faDbqaaiabdsgaKjabdsha0baacqGH9aqpcqWGZbWCdaWgaaWcbaGaeeyBa0MaeeizaqMaeeyBa0MaeGOmaidabeaakiabgkHiTGGaciab=r7aKnaaBaaaleaacqqGTbqBcqqGKbazcqqGTbqBcqaIYaGmaeqaaOGaei4waSLaemyBa0MaemizaqMaemyBa0MaeGOmaiJaeiyxa0Laey4kaSIae8xTdu2aaSbaaSqaaiabb2gaTjabbsgaKjabb2gaTjabikdaYaqabaGcdaWcaaqaaiabcUfaBjabbchaWjabiwda1iabiodaZiabcQcaQiabcIcaOiabdsha0jabgkHiTiab=r8a0naaBaaaleaacqaIXaqmaeqaaOGaeiykaKIaeiyxa01aaWbaaSqabeaacqWGUbGBaaaakeaacqGGBbWwcqqGWbaCcqaI1aqncqaIZaWmcqGGQaGkcqGGOaakcqWG0baDcqGHsislcqWFepaDdaWgaaWcbaGaeGymaedabeaakiabcMcaPiabc2faDnaaCaaaleqabaGaemOBa4gaaOGaey4kaSIaem4saS0aaWbaaSqabeaacqWGUbGBaaaaaOGaeiilaWIaaCzcaiaaxMaadaqadaqaaiabiwda1iabbkgaIbGaayjkaiaawMcaaaaa@7F3D@

where the transcription rate of p53 is invariant and leads to a constant mRNA steady state and p53*(*t *- *τ*_1_) is the activated p53 protein level, which is phosphorylated by the phosphorylated ATM. The effect of the activated p53 on *mdm2 *is delayed by *τ*_1_. Since this protein activation part is unknown, the effect of p53 on *mdm2 *is hidden in the measurements and the estimated Jacobian will be effected accordingly. The unknown kinetics includes negative feedback interactions between p53 and Mdm2 proteins and the stochastic dynamics of double-strand break complexes – see [14] for more details. The true Jacobian is simply given by

F=[−δp5300−δmdm2]=[−0.0200−0.02],     (6)
 MathType@MTEF@5@5@+=feaafiart1ev1aaatCvAUfKttLearuWrP9MDH5MBPbIqV92AaeXatLxBI9gBaebbnrfifHhDYfgasaacH8akY=wiFfYdH8Gipec8Eeeu0xXdbba9frFj0=OqFfea0dXdd9vqai=hGuQ8kuc9pgc9s8qqaq=dirpe0xb9q8qiLsFr0=vr0=vr0dc8meaabaqaciaacaGaaeqabaqabeGadaaakeaacqWGgbGrcqGH9aqpdaWadaqaauaabeqaciaaaeaacqGHsisliiGacqWF0oazdaWgaaWcbaGaeeiCaaNaeGynauJaeG4mamdabeaaaOqaaiabicdaWaqaaiabicdaWaqaaiabgkHiTiab=r7aKnaaBaaaleaacqqGTbqBcqqGKbazcqqGTbqBcqaIYaGmaeqaaaaaaOGaay5waiaaw2faaiabg2da9maadmaabaqbaeqabiGaaaqaaiabgkHiTiabicdaWiabc6caUiabicdaWiabikdaYaqaaiabicdaWaqaaiabicdaWaqaaiabgkHiTiabicdaWiabc6caUiabicdaWiabikdaYaaaaiaawUfacaGLDbaacqGGSaalcaWLjaGaaCzcamaabmaabaGaeGOnaydacaGLOaGaayzkaaaaaa@5356@

where the numbers are given in [14].

**Figure 3:**
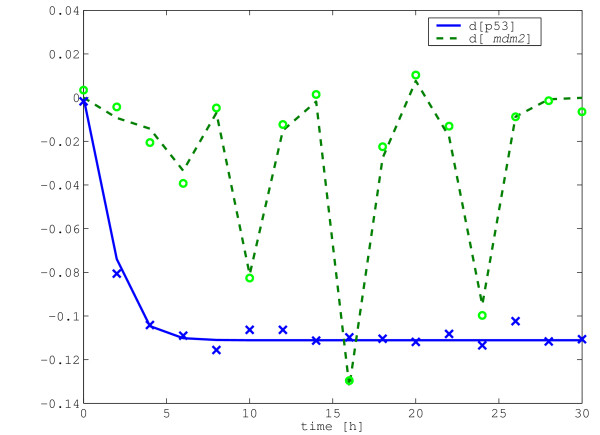
**The measurements of the perturbed p53 and *mdm2 *gene expression levels**. An example of the measurements for the perturbed p53 and *mdm2 *gene expression levels are shown. The data are generated from the model suggested in [14]. The true perturbed gene expression levels are shown in lines, and the corresponding measurements are the cross for p53 and the circle for *mdm2*, respectively.

The perturbation level on p53 is negative 10%, the measurement sampling time is 2 hours and white noise is added to the measurement data. Note that the true states converge to a steady state after around 16 samples, i.e. 30 hours. The true and the measured values of the perturbed gene expression levels are shown in Figure 3. The estimation results are shown in Table 4. In most cases, the average errors for all measures produced by the CTLS are the smallest. Similarly to the previous example, the error reduction stops after the number of data points reaches around 8. Hence, further reductions in the estimation error for this example would require the application of more accurate detection methodologies. Since there are some ignored kinetics, even with virtually no measurement error data the Jacobian still gives some connections between p53 and *mdm2*. Then, we may conclude that there are some additional kinetics, which have not been discovered yet, and this may motivate further experiments to elucidate these hidden regulatory mechanisms.

**Table 4:** p53 and mdm2 mRNA expression model: white noise with neglected kinetics

Samplings per Experiment	Algorithms	*ε*_*M*_		*ε*_*S*_		*ε*_*F*_	
		Mean	STD	Mean	STD	Mean	STD
4	LS	1.34	0.34	1.01	0.14	0.04	0.01
	TLS	1.34	0.34	1.01	0.14	0.04	0.01
	CTLS	1.34	0.34	1.01	0.14	0.04	0.01

8	LS	0.95	0.27	0.50	0.02	0.03	0.01
	TLS	25.03	196.73	1.01	0.15	1.06	8.42
	CTLS	0.44	0.12	0.50	0.00	0.02	0.00

12	LS	0.61	0.08	0.50	0.00	0.02	0.00
	TLS	47.53	241.86	0.92	0.31	2.49	13.07
	CTLS	0.49	0.06	0.50	0.02	0.02	0.00

16	LS	0.44	0.06	0.50	0.00	0.02	0.00
	TLS	50.96	833.28	1.02	0.20	3.11	50.06
	CTLS	0.49	0.05	0.50	0.02	0.02	0.00

The table shows the error comparisons in terms of the mean and the standard deviation (STD) for different number of data for each method based on 1000 Monte-Carlo Simulations. The measurements are taken every 2 hours and the states converge to steady states around the 16-th sample. *ε*_*M *_is the sum of two tems, i.e (1/*N*_1_) Σ |*α*_*i j*_| and (1/*N*_2_) Σ |*β*_*i j*_|, where *α*_*i j *_and *β*_*i j *_are the relative magnitude errors in the non-zero and zero elements of the true Jacobian, respectively, and *N*_1 _and *N*_2 _are the number of non-zero and zero elements in the true Jacobian, respectively. *ε*_*S *_is given by (1/*n*^2^) Σ |sign (f^
 MathType@MTEF@5@5@+=feaafiart1ev1aaatCvAUfKttLearuWrP9MDH5MBPbIqV92AaeXatLxBI9gBaebbnrfifHhDYfgasaacH8akY=wiFfYdH8Gipec8Eeeu0xXdbba9frFj0=OqFfea0dXdd9vqai=hGuQ8kuc9pgc9s8qqaq=dirpe0xb9q8qiLsFr0=vr0=vr0dc8meaabaqaciaacaGaaeqabaqabeGadaaakeaacuWGMbGzgaqcaaaa@2E11@_*i j*_) - sign (*f*_*i j*_)|, i.e. the average sign differences, where f^
 MathType@MTEF@5@5@+=feaafiart1ev1aaatCvAUfKttLearuWrP9MDH5MBPbIqV92AaeXatLxBI9gBaebbnrfifHhDYfgasaacH8akY=wiFfYdH8Gipec8Eeeu0xXdbba9frFj0=OqFfea0dXdd9vqai=hGuQ8kuc9pgc9s8qqaq=dirpe0xb9q8qiLsFr0=vr0=vr0dc8meaabaqaciaacaGaaeqabaqabeGadaaakeaacuWGMbGzgaqcaaaa@2E11@_*i j *_and *f*_*i j *_are the (*i*-th row, *j*-th column) elements of the estimated and the true Jacobian, respectively. *ε*_*F *_is the Frobenius norm of the difference between the estimated and the true Jacobian, i.e. ||F^
 MathType@MTEF@5@5@+=feaafiart1ev1aaatCvAUfKttLearuWrP9MDH5MBPbIqV92AaeXatLxBI9gBaebbnrfifHhDYfgasaacH8akY=wiFfYdH8Gipec8Eeeu0xXdbba9frFj0=OqFfea0dXdd9vqai=hGuQ8kuc9pgc9s8qqaq=dirpe0xb9q8qiLsFr0=vr0=vr0dc8meaabaqaciaacaGaaeqabaqabeGadaaakeaacuWGgbGrgaqcaaaa@2DD1@ - *F*||_*F*_.

### *IL6 *and *IL12 *messenger RNA expression

**Figure 4:**
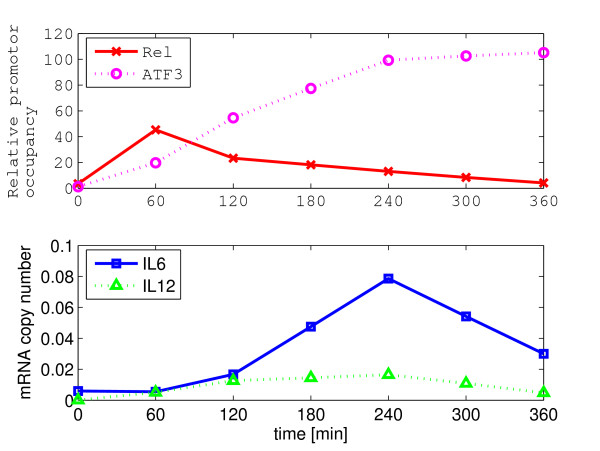
**The measurements of the prominent network in the innate immune system**. The measurements of Rel, ATF3, I16, and I112 are taken from [15]. The actual data in [15] are measured at 0, 60, 120, 240, 360 minutes. To make the measurements equally spaced in time, the data at 180 and 300 minutes are interpolated.

Proper regulation of the innate immune system is crucial for host survival, and is mediated, in part, by cytokines that are secreted by macrophages. In particular, breakdown of immune system regulatory mechanisms can lead to inflammatory disease. Immune system control is extraordinarily complex, making it an obvious candidate for investigation using Systems Biology approaches. The biochemical network through which interleukin (IL)-6 and IL- 12b interact with activating transcription factor 3 (ATF3) and Rel (a component of NF-*κ*B) forms an important part of the innate immune system response [15]. A kinetic model for the expression of *IL6 *mRNA by *ATF3 *and *Rel *was recently proposed in [15] as follows:

d[Il6]dt=−1τ[Il6]+1τ(1+e−βRel[Rel]−βATF3[ATF3])     (7)
 MathType@MTEF@5@5@+=feaafiart1ev1aaatCvAUfKttLearuWrP9MDH5MBPbIqV92AaeXatLxBI9gBamXvP5wqSXMqHnxAJn0BKvguHDwzZbqegyvzYrwyUfgarqqtubsr4rNCHbGeaGqipGI8VfYxH8qipiYdHaVhbbf9v8qqaqFr0xc9pk0xbba9q8WqFfeaY=biLkVcLq=JHqVepeea0=as0db9vqpepesP0xe9Fve9Fve9GapdbaqaaeGacaGaaiaabeqaamqadiabaaGcbaWaaSaaaeaacqWGKbazcqGGBbWwcqWGjbqscqWGSbaBcqaI2aGncqGGDbqxaeaacqWGKbazcqWG0baDaaGaeyypa0JaeyOeI0YaaSaaaeaacqaIXaqmaeaaiiGacqWFepaDaaGaei4waSLaemysaKKaemiBaWMaeGOnayJaeiyxa0Laey4kaSYaaSaaaeaacqaIXaqmaeaacqWFepaDcqGGOaakcqaIXaqmcqGHRaWkcqWGLbqzdaahaaWcbeqaaiabgkHiTiab=j7aInaaBaaameaacqqGsbGucqqGLbqzcqqGSbaBaeqaaSGaei4waSfcbiGae4NuaiLae4xzauMaemiBaWMaeiyxa0LaeyOeI0Iae8NSdi2aaSbaaWqaaiabbgeabjabbsfaujabbAeagjabiodaZaqabaWccqGGBbWwcqWGbbqqcqWGubavcqWGgbGrcqaIZaWmcqGGDbqxaaGccqGGPaqkaaGaaCzcaiaaxMaadaqadaqaaiabiEda3aGaayjkaiaawMcaaaaa@733A@

where *τ *= 600/ln(2), *β*_Rel _= 7.8, *β*_ATF3 _= -4.9, [*Il6*] is the *Il6 *mRNA expression level, and [*Rel*] and [*ATF3*] are the level of *Rel *and *ATF3*, respectively. The second part in the right hand side of the kinetic equation is a sigmoidal function that incorporates lower and upper bounds on *Il6 *expression, *τ *is given by *T*^1/2^/ln(2) and *T*^1/2 ^is a typical mRNA half-life in mammalian cells, and *β*_Rel _and *β*_ATF3 _represent the relative contributions of *Rel *and *ATF3 *in the levels of *Il6 *transcription, respectively. In [15], this kinetic model was developed to match the experimental data shown in Figure 4 using a least squares regression. Similarly, a kinetic model for *IL12 *is given by

d[Il12]dt=−1τ[Il12]+1τ(1+e−βRel[Rel]−βATF3[ATF3])     (8)
 MathType@MTEF@5@5@+=feaafiart1ev1aaatCvAUfKttLearuWrP9MDH5MBPbIqV92AaeXatLxBI9gBaebbnrfifHhDYfgasaacH8akY=wiFfYdH8Gipec8Eeeu0xXdbba9frFj0=OqFfea0dXdd9vqai=hGuQ8kuc9pgc9s8qqaq=dirpe0xb9q8qiLsFr0=vr0=vr0dc8meaabaqaciaacaGaaeqabaqabeGadaaakeaadaWcaaqaaiabdsgaKjabcUfaBjabdMeajjabdYgaSjabigdaXiabikdaYiabc2faDbqaaiabdsgaKjabdsha0baacqGH9aqpcqGHsisldaWcaaqaaiabigdaXaqaaGGaciab=r8a0baacqGGBbWwcqWGjbqscqWGSbaBcqaIXaqmcqaIYaGmcqGGDbqxcqGHRaWkdaWcaaqaaiabigdaXaqaaiab=r8a0jabcIcaOiabigdaXiabgUcaRiabdwgaLnaaCaaaleqabaGaeyOeI0Iae8NSdi2aaSbaaWqaaiabbkfasjabbwgaLjabbYgaSbqabaWccqGGBbWwieGacqGFsbGucqGFLbqzcqWGSbaBcqGGDbqxcqGHsislcqWFYoGydaWgaaadbaacbaGae0xqaeKae0hvaqLae0NrayKaeG4mamdabeaaliabcUfaBjabdgeabjabdsfaujabdAeagjabiodaZiabc2faDbaakiabcMcaPaaacaWLjaGaaCzcamaabmaabaGaeGioaGdacaGLOaGaayzkaaaaaa@6A85@

where *τ *= 600/ln(2), *β*_Rel _= 18.5, *β*_ATF3 _= -9.6, and [*IL12*] is the level of *Il12 *mRNA expression. Similar interpretations to those for *Il6 *can be applied to this equation. Unlike with the previous example, since this is a model based on real data from a partially understood biochemical network, the true Jacobian is unknown, and therefore the estimation error cannot be evaluated explicitly. However, using the proposed kinetic models we can obtain the following ratio:

∂(d[Il6]/dt)∂[Rel][∂(d[Il6]/dt)∂[ATF3]]−1=∂[ATF3]∂[Rel]=βRelβATF3=7.8−4.9≈−1.59,     (9)
 MathType@MTEF@5@5@+=feaafiart1ev1aaatCvAUfKttLearuWrP9MDH5MBPbIqV92AaeXatLxBI9gBaebbnrfifHhDYfgasaacH8akY=wiFfYdH8Gipec8Eeeu0xXdbba9frFj0=OqFfea0dXdd9vqai=hGuQ8kuc9pgc9s8qqaq=dirpe0xb9q8qiLsFr0=vr0=vr0dc8meaabaqaciaacaGaaeqabaqabeGadaaakeaadaWcaaqaaiabgkGi2oaabmaabaGaemizaqMaei4waSLaemysaKKaemiBaWMaeGOnayJaeiyxa0Laei4la8IaemizaqMaemiDaqhacaGLOaGaayzkaaaabaGaeyOaIyRaei4waSfcbiGae8NuaiLae8xzauMaemiBaWMaeiyxa0faamaadmaabaWaaSaaaeaacqGHciITdaqadaqaaiabdsgaKjabcUfaBjabdMeajjabdYgaSjabiAda2iabc2faDjabc+caViabdsgaKjabdsha0bGaayjkaiaawMcaaaqaaiabgkGi2kabcUfaBjabdgeabjabdsfaujabdAeagjabiodaZiabc2faDbaaaiaawUfacaGLDbaadaahaaWcbeqaaiabgkHiTiabigdaXaaakiabg2da9maalaaabaGaeyOaIyRaei4waSLaemyqaeKaemivaqLaemOrayKaeG4mamJaeiyxa0fabaGaeyOaIyRaei4waSLae8NuaiLae8xzauMaemiBaWMaeiyxa0faaiabg2da9maalaaabaacciGae4NSdi2aaSbaaSqaaiabbkfasjabbwgaLjabbYgaSbqabaaakeaacqGFYoGydaWgaaWcbaGaeeyqaeKaeeivaqLaeeOrayKaeG4mamdabeaaaaGccqGH9aqpdaWcaaqaaiabiEda3iabc6caUiabiIda4aqaaiabgkHiTiabisda0iabc6caUiabiMda5aaacqGHijYUcqGHsislcqaIXaqmcqGGUaGlcqaI1aqncqaI5aqocqGGSaalcaWLjaGaaCzcamaabmaabaGaeGyoaKdacaGLOaGaayzkaaaaaa@8D33@

and therefore we can partially validate the Jacobian estimation from the data against the proposed model by checking the value of this ratio. The equivalent ratio for the case of *IL12 *is -1.93. Note that in the context of this example, the negative sign of this value is crucial since it corresponds to a negative feedback role for *ATF3*, which was the main finding presented in [15].

In [15], to obtain the data shown in Figure 4 wild type mice were stimulated (or perturbed) by 10 ng ml^-1 ^lipopolysaccharide (LPS). The data was sampled at intervals of 10 minutes but the original data at 180 and 300 minutes were not given, hence, they are interpolated for our study to make all data equally spaced in time. Of course, the measurement data will definitely include some noise and the direct calculation of the Jacobian using the conventional least squares may therefore produce biased/inaccurate results. Note that since the number of states is 3, the number of perturbations is 1, and the number of data points for each state is 7, there is relatively little data with which to accurately estimate the Jacobian for this particular example. However, using the various least squares algorithms, we tried to extract the maximum amount of information from the given set of experimental data. In addition, note that since the equilibrium point is not given, the measurements we have are not relative measurements Δx˜
 MathType@MTEF@5@5@+=feaafiart1ev1aaatCvAUfKttLearuWrP9MDH5MBPbIqV92AaeXatLxBI9gBaebbnrfifHhDYfgasaacH8akY=wiFfYdH8Gipec8Eeeu0xXdbba9frFj0=OqFfea0dXdd9vqai=hGuQ8kuc9pgc9s8qqaq=dirpe0xb9q8qiLsFr0=vr0=vr0dc8meaabaqaciaacaGaaeqabaqabeGadaaakeaacuWG4baEgaacaaaa@2E34@_*k *_but absolute measurements x˜
 MathType@MTEF@5@5@+=feaafiart1ev1aaatCvAUfKttLearuWrP9MDH5MBPbIqV92AaeXatLxBI9gBaebbnrfifHhDYfgasaacH8akY=wiFfYdH8Gipec8Eeeu0xXdbba9frFj0=OqFfea0dXdd9vqai=hGuQ8kuc9pgc9s8qqaq=dirpe0xb9q8qiLsFr0=vr0=vr0dc8meaabaqaciaacaGaaeqabaqabeGadaaakeaacuWG4baEgaacaaaa@2E34@_*k*_. This presents no difficulty, however, since in the framework of [1], the problem formulation to estimate the Jacobian using x˜
 MathType@MTEF@5@5@+=feaafiart1ev1aaatCvAUfKttLearuWrP9MDH5MBPbIqV92AaeXatLxBI9gBaebbnrfifHhDYfgasaacH8akY=wiFfYdH8Gipec8Eeeu0xXdbba9frFj0=OqFfea0dXdd9vqai=hGuQ8kuc9pgc9s8qqaq=dirpe0xb9q8qiLsFr0=vr0=vr0dc8meaabaqaciaacaGaaeqabaqabeGadaaakeaacuWG4baEgaacaaaa@2E34@_*k *_is exactly the same as the one for Δx˜
 MathType@MTEF@5@5@+=feaafiart1ev1aaatCvAUfKttLearuWrP9MDH5MBPbIqV92AaeXatLxBI9gBaebbnrfifHhDYfgasaacH8akY=wiFfYdH8Gipec8Eeeu0xXdbba9frFj0=OqFfea0dXdd9vqai=hGuQ8kuc9pgc9s8qqaq=dirpe0xb9q8qiLsFr0=vr0=vr0dc8meaabaqaciaacaGaaeqabaqabeGadaaakeaacuWG4baEgaacaaaa@2E34@_*k *_– see [1] for more details. For *Il6 *the key result for this example is that the standard least squares algorithm gives the wrong (positive) sign for the ratio defined above, whereas the more advanced algorithms give the correct sign. The correct ratio of *Rel *and *ATF3 *to *Il6 *is -1.59 and the estimated values computed with the LS, TLS, and CTLS algorithms are 1.43, -3.73, and -6.35, respectively. Thus, only by using the TLS or CTLS algorithms can the negative regulation effect of ATF3 proposed in [15] be confirmed from the noisy data presented in the paper. For *Il12*, the ratio calculated from each method, i.e., LS, TLS, and CTLS, is -4.53, -2.46, and -1.98, respectively. Therefore, in this case all three algorithms predict the negative regulation role of ATF3 correctly. However, the ratio computed from the CTLS, -1.98, is by far the closest to the true value (-1.93) predicted by the model. The improved parameterization achieved using the CTLS algorithm means that additional factors, in particular API and perhaps chromatin remodelling factors, can be added to the model with only limited and targeted new biological data.

## Conclusion

We have considered the problem of identifying the dynamic interactions in biochemical networks from noisy data. Since time-series measurements of, for example, concentrations and expression profiles in gene networks, are almost guaranteed to be corrupted by significant levels of noise, algorithms are required which explicitly take this noise into account when computing estimates of quantitative interactions in biochemical networks. The TLS and CTLS algorithms are extensions of the widely used least squares approach which optimally deal with the presence of uncorrelated and correlated noise in the measurements, respectively. Since noise in time-series measurements from biological experiments are generally correlated, the CTLS approach is ideally suited to estimation problems of this type.

The superior performance of the CTLS method in identifying network interactions was demonstrated on three examples: a genetic network containing four genes, a high fidelity p53 and *mdm2 *interaction network, and a recently proposed kinetic model for interleukin (IL)-6 and (IL)-12b messenger RNA expression as a function of ATF3 and NF-*κ*B promoter binding. For the first example, the CTLS has significantly reduced the errors in the estimation of the Jacobian for the gene network. For the second, the CTLS shows similarly superior performance over the other least-squares methods to estimate the Jacobian from the measurements of a high fidelity gene network with neglected kinetics. For the third, it has allowed the correct identification, from noisy data, of the negative regulation of (IL)-6 and (IL)-12b by ATF3. The ability to take into account errors from various noise sources when identifying biochemical networks is valuable in informing decisions about the optimal numbers of data measurements that are required. While the use of very few data points generates huge errors, the errors do not decrease monotonically with much larger numbers of points. Thus, the calculations presented in this paper provide a rational basis for the design of experiments, in particular regarding the required frequency and accuracy of sampling. This is a very important issue in practice, since for some experiments there will be a practical upper limit on the number of data points per experiment (e.g. some measurements may take a certain amount of time to make; or if a measurement uses 5% of a starting culture which changes properties if scaled by more than 2-fold, one can only ever obtain 10 measurements). Secondly, the number of data points is often a trade-off with accuracy of the measurements. Typically, one would schedule a single day to do an experiment and then the choice is often between making more, noisier measurements or fewer, more accurate ones (since it is usually impossible to make conditions on two different days exactly the same.)

The excellent performance of the CTLS method compared to LS and TLS approaches (or the implicit assumption of perfect data) under the wide range of conditions tested here – including different levels of noise, different numbers of data points, and with drift – suggests that its application will enable better identification and modelling of biochemical networks. In the future, explicit analysis with the CTLS method is likely to increase the number of parameters that can be included in a model, even where there is limited knowledge of the noise levels and their source.

## Methods

### Problem Formulation

In general, the dynamics of many biochemical networks can be modelled as a nonlinear differential equation [1]

x˙
 MathType@MTEF@5@5@+=feaafiart1ev1aaatCvAUfKttLearuWrP9MDH5MBPbIqV92AaeXatLxBI9gBaebbnrfifHhDYfgasaacH8akY=wiFfYdH8Gipec8Eeeu0xXdbba9frFj0=OqFfea0dXdd9vqai=hGuQ8kuc9pgc9s8qqaq=dirpe0xb9q8qiLsFr0=vr0=vr0dc8meaabaqaciaacaGaaeqabaqabeGadaaakeaacuWG4baEgaGaaaaa@2E2E@(*t*) = *f *(*x*(*t*))     (10)

where x˙
 MathType@MTEF@5@5@+=feaafiart1ev1aaatCvAUfKttLearuWrP9MDH5MBPbIqV92AaeXatLxBI9gBaebbnrfifHhDYfgasaacH8akY=wiFfYdH8Gipec8Eeeu0xXdbba9frFj0=OqFfea0dXdd9vqai=hGuQ8kuc9pgc9s8qqaq=dirpe0xb9q8qiLsFr0=vr0=vr0dc8meaabaqaciaacaGaaeqabaqabeGadaaakeaacuWG4baEgaGaaaaa@2E2E@(*t*) is the time derivative of *x*(*t*), i.e. *dx *(*t*)/*dt*, and *x*(*t*) is an element of ℝ^*n *^where ℝ^*n *^is the real *n*-dimensional space. Note that the symbol *x *is used for two purposes in this paper, one for the unknown in the linear equation *Ax *= *b *and the other for the state of an ordinary differential equation. To distinguish between them, the state vector of the ordinary differential equation will always be written as *x*(*t*), i.e. as a function of time. In the above differential equation, *f *(·) is a nonlinear function, which satisfies the conditions for the existence and uniqueness of the solution of the ordinary differential equation. In biochemical networks *f *(·) is often also a function of some experimentally adjustable parameters such as kinetic rate constants or gene transcription rates [1].

If the above system is perturbed at an equilibrium point, *x*_0_, that satisfies *f *(*x*_0_) = 0, then the state around the equilibrium point is also perturbed by Δ*x*, and satisfies the following linear ordinary differential equation:

Δx˙
 MathType@MTEF@5@5@+=feaafiart1ev1aaatCvAUfKttLearuWrP9MDH5MBPbIqV92AaeXatLxBI9gBaebbnrfifHhDYfgasaacH8akY=wiFfYdH8Gipec8Eeeu0xXdbba9frFj0=OqFfea0dXdd9vqai=hGuQ8kuc9pgc9s8qqaq=dirpe0xb9q8qiLsFr0=vr0=vr0dc8meaabaqaciaacaGaaeqabaqabeGadaaakeaacuWG4baEgaGaaaaa@2E2E@ (*t*) = *F *Δ *x*(*t*) + *u*(*t*).     (11)

In the following, *u*(*t*) is assumed to be a constant, however, all results also hold in the case of a time-varying *u *(*t*) [1]. From the above equation, it is clear that the matrix *F *reveals the relations between each state in *x *(*t*) around the equilibrium point. The main purpose of this paper is to provide a more efficient and reliable method for estimating the matrix *F*, known as the Jacobian of *f *(*x*(*t*)) at *x *(*t*) = *x*_0_, when the measurement data are effected by noise. Since the measurement data are recorded at discrete time intervals, we reformulate the continuous time system as a discrete time system given by

Δ*x*_*k *+ 1 _= Φ Δ*x*_*k *_+ *u *    (12)

where Δ*x*_*k *+ 1 _and Δ*x*_*k *_are the sampled versions of the corresponding states in the continuous system and Φ is an *n *× *n *matrix in ℝ^*n *× *n*^. Whenever Φ is determined, the original *F *can be recovered using the following relation [1]:

F=1ΔTlog⁡(Φ)     (13)
 MathType@MTEF@5@5@+=feaafiart1ev1aaatCvAUfKttLearuWrP9MDH5MBPbIqV92AaeXatLxBI9gBaebbnrfifHhDYfgasaacH8akY=wiFfYdH8Gipec8Eeeu0xXdbba9frFj0=OqFfea0dXdd9vqai=hGuQ8kuc9pgc9s8qqaq=dirpe0xb9q8qiLsFr0=vr0=vr0dc8meaabaqaciaacaGaaeqabaqabeGadaaakeaacqWGgbGrcqGH9aqpdaWcaaqaaiabigdaXaqaaiabfs5aejabdsfaubaacyGGSbaBcqGGVbWBcqGGNbWzcqGGOaakcqqHMoGrcqGGPaqkcaWLjaGaaCzcamaabmaabaGaeGymaeJaeG4mamdacaGLOaGaayzkaaaaaa@3E59@

where Δ*T *is the sampling time and log(Φ) is the solution of *e*^*X *^= Φ. Note, however, that when Φ is very poorly estimated, it could happen that log(Φ) becomes a complex matrix. To avoid this biologically meaningless result, the following approximation can be used [5]:

F=2ΔT(Φ−In)(Φ+In)−1     (14)
 MathType@MTEF@5@5@+=feaafiart1ev1aaatCvAUfKttLearuWrP9MDH5MBPbIqV92AaeXatLxBI9gBaebbnrfifHhDYfgasaacH8akY=wiFfYdH8Gipec8Eeeu0xXdbba9frFj0=OqFfea0dXdd9vqai=hGuQ8kuc9pgc9s8qqaq=dirpe0xb9q8qiLsFr0=vr0=vr0dc8meaabaqaciaacaGaaeqabaqabeGadaaakeaacqWGgbGrcqGH9aqpdaWcaaqaaiabikdaYaqaaiabfs5aejabdsfaubaacqGGOaakcqqHMoGrcqGHsislcqWGjbqsdaWgaaWcbaGaemOBa4gabeaakiabcMcaPiabcIcaOiabfA6agjabgUcaRiabdMeajnaaBaaaleaacqWGUbGBaeqaaOGaeiykaKYaaWbaaSqabeaacqGHsislcqaIXaqmaaGccaWLjaGaaCzcamaabmaabaGaeGymaeJaeGinaqdacaGLOaGaayzkaaaaaa@46BA@

where *I*_*n *_is the *n *× *n *identity matrix. Even with this approximation, however, there could exist numerically ill-conditioned problems if Φ + *I*_*n *_is close to singular and thereby not invertible. In such situations the following Euler approximation can be used instead [1]:

F=1ΔT(Φ−In),     (15)
 MathType@MTEF@5@5@+=feaafiart1ev1aaatCvAUfKttLearuWrP9MDH5MBPbIqV92AaeXatLxBI9gBaebbnrfifHhDYfgasaacH8akY=wiFfYdH8Gipec8Eeeu0xXdbba9frFj0=OqFfea0dXdd9vqai=hGuQ8kuc9pgc9s8qqaq=dirpe0xb9q8qiLsFr0=vr0=vr0dc8meaabaqaciaacaGaaeqabaqabeGadaaakeaacqWGgbGrcqGH9aqpdaWcaaqaaiabigdaXaqaaiabfs5aejabdsfaubaacqGGOaakcqqHMoGrcqGHsislcqWGjbqsdaWgaaWcbaGaemOBa4gabeaakiabcMcaPiabcYcaSiaaxMaacaWLjaWaaeWaaeaacqaIXaqmcqaI1aqnaiaawIcacaGLPaaaaaa@3EC2@

although in this case the Jacobian *F *will be very sensitive to the magnitude of Δ*T*. Clearly, however, if the transformations using (13) and (14) fail then Φ is not a very good estimate and, hence, the resulting *F *from (15) cannot be expected to be close to the true Jacobian.

Let us consider *L *noisy measurements of Δ*x*_*k*_. Then we have

Δx˜
 MathType@MTEF@5@5@+=feaafiart1ev1aaatCvAUfKttLearuWrP9MDH5MBPbIqV92AaeXatLxBI9gBaebbnrfifHhDYfgasaacH8akY=wiFfYdH8Gipec8Eeeu0xXdbba9frFj0=OqFfea0dXdd9vqai=hGuQ8kuc9pgc9s8qqaq=dirpe0xb9q8qiLsFr0=vr0=vr0dc8meaabaqaciaacaGaaeqabaqabeGadaaakeaacuWG4baEgaacaaaa@2E34@_*k *_= Δ*x*_*k *_+ *v*_*k *_for *k *= 1, 2, ..., *L *    (16)

where *v*_*k *_is a zero-mean white noise vector in ℝ^*n*^. That is, *E *(*v*_*k*_) = 0 and *E *(*v*_*i *_vjT
 MathType@MTEF@5@5@+=feaafiart1ev1aaatCvAUfKttLearuWrP9MDH5MBPbIqV92AaeXatLxBI9gBaebbnrfifHhDYfgasaacH8akY=wiFfYdH8Gipec8Eeeu0xXdbba9frFj0=OqFfea0dXdd9vqai=hGuQ8kuc9pgc9s8qqaq=dirpe0xb9q8qiLsFr0=vr0=vr0dc8meaabaqaciaacaGaaeqabaqabeGadaaakeaacqWG2bGDdaqhaaWcbaGaemOAaOgabaGaemivaqfaaaaa@30DC@) = 0 for *i *≠ *j *where *E *(·) is the Expectation. The Expectation is defined through a corresponding probability density function, i.e. *E *(*v*_*k*_) = ∫_Ω _*v*_*k *_*p*(*ω*) *d*Ω where *p *(*ω*) is the probability density function and Ω is the sample space. In simple terms, *E *(*v*_*k*_) can be regarded as the average of *v*_*k *_and *E *(vk2
 MathType@MTEF@5@5@+=feaafiart1ev1aaatCvAUfKttLearuWrP9MDH5MBPbIqV92AaeXatLxBI9gBaebbnrfifHhDYfgasaacH8akY=wiFfYdH8Gipec8Eeeu0xXdbba9frFj0=OqFfea0dXdd9vqai=hGuQ8kuc9pgc9s8qqaq=dirpe0xb9q8qiLsFr0=vr0=vr0dc8meaabaqaciaacaGaaeqabaqabeGadaaakeaacqWG2bGDdaqhaaWcbaGaem4AaSgabaGaeGOmaidaaaaa@309F@) as the variance of *v*_*k*_.

Assuming that the number of measurements, *L*, is greater than *n *+ 2, from the relations given in (12) and (16) we have

Δ*x*_*k *_= Φ Δ*x*_*k *- 1 _+ *u *= Φ Δ x˜
 MathType@MTEF@5@5@+=feaafiart1ev1aaatCvAUfKttLearuWrP9MDH5MBPbIqV92AaeXatLxBI9gBaebbnrfifHhDYfgasaacH8akY=wiFfYdH8Gipec8Eeeu0xXdbba9frFj0=OqFfea0dXdd9vqai=hGuQ8kuc9pgc9s8qqaq=dirpe0xb9q8qiLsFr0=vr0=vr0dc8meaabaqaciaacaGaaeqabaqabeGadaaakeaacuWG4baEgaacaaaa@2E34@_*k *- 1 _+ *u *- Φ*v*_*k *- 1 _    (17)

for *k *= 1, 2, ..., *L*. However, since the true values of Δ*x*_*k *_are not known, the left hand side of (17) must be replaced by the corresponding measured values:

Δx˜
 MathType@MTEF@5@5@+=feaafiart1ev1aaatCvAUfKttLearuWrP9MDH5MBPbIqV92AaeXatLxBI9gBaebbnrfifHhDYfgasaacH8akY=wiFfYdH8Gipec8Eeeu0xXdbba9frFj0=OqFfea0dXdd9vqai=hGuQ8kuc9pgc9s8qqaq=dirpe0xb9q8qiLsFr0=vr0=vr0dc8meaabaqaciaacaGaaeqabaqabeGadaaakeaacuWG4baEgaacaaaa@2E34@_*k *_- *v*_*k *_= Φ Δx˜
 MathType@MTEF@5@5@+=feaafiart1ev1aaatCvAUfKttLearuWrP9MDH5MBPbIqV92AaeXatLxBI9gBaebbnrfifHhDYfgasaacH8akY=wiFfYdH8Gipec8Eeeu0xXdbba9frFj0=OqFfea0dXdd9vqai=hGuQ8kuc9pgc9s8qqaq=dirpe0xb9q8qiLsFr0=vr0=vr0dc8meaabaqaciaacaGaaeqabaqabeGadaaakeaacuWG4baEgaacaaaa@2E34@_*k *- 1 _+ *u *- Φ *v*_*k *- 1 _    (18)

for *k *= 1, 2, ..., *L*. The above can be written in a matrix form as follows:

ΔX˜(2..L)+V(2..L)=[Φ  u]{[ΔX˜(1..(L−1))11×(L−1)]+[V(1..(L−1))01×(L−1)]}     (19)
 MathType@MTEF@5@5@+=feaafiart1ev1aaatCvAUfKttLearuWrP9MDH5MBPbIqV92AaeXatLxBI9gBaebbnrfifHhDYfgasaacH8akY=wiFfYdH8Gipec8Eeeu0xXdbba9frFj0=OqFfea0dXdd9vqai=hGuQ8kuc9pgc9s8qqaq=dirpe0xb9q8qiLsFr0=vr0=vr0dc8meaabaqaciaacaGaaeqabaqabeGadaaakeaacqqHuoarcuWGybawgaacamaaBaaaleaacqGGOaakcqaIYaGmcqGGUaGlcqGGUaGlcqWGmbatcqGGPaqkaeqaaOGaey4kaSIaemOvay1aaSbaaSqaaiabcIcaOiabikdaYiabc6caUiabc6caUiabdYeamjabcMcaPaqabaGccqGH9aqpcqGGBbWwcqqHMoGrcqqGGaaicqqGGaaicqWG1bqDcqGGDbqxdaGadeqaamaadmaabaqbaeqabiqaaaqaaiabfs5aejqbdIfayzaaiaWaaSbaaSqaaiabcIcaOiabigdaXiabc6caUiabc6caUiabcIcaOiabdYeamjabgkHiTiabigdaXiabcMcaPiabcMcaPaqabaaakeaaieqacqWFXaqmdaWgaaWcbaGaeGymaeJaey41aqRaeiikaGIaemitaWKaeyOeI0IaeGymaeJaeiykaKcabeaaaaaakiaawUfacaGLDbaacqGHRaWkdaWadaqaauaabeqaceaaaeaacqWGwbGvdaWgaaWcbaGaeiikaGIaeGymaeJaeiOla4IaeiOla4IaeiikaGIaemitaWKaeyOeI0IaeGymaeJaeiykaKIaeiykaKcabeaaaOqaaiab=bdaWmaaBaaaleaacqaIXaqmcqGHxdaTcqGGOaakcqWGmbatcqGHsislcqaIXaqmcqGGPaqkaeqaaaaaaOGaay5waiaaw2faaaGaay5Eaiaaw2haaiaaxMaacaWLjaWaaeWaaeaacqaIXaqmcqaI5aqoaiaawIcacaGLPaaaaaa@78FA@

where **1**_1 × (*L *- 1) _and **0**_1 × (*L *- 1) _are 1 × (*L *- 1) vectors with elements of 1 or 0, and

ΔX˜(i..j)=[Δx˜iΔx˜i+1…Δx˜j−1Δx˜j],     (20a)
 MathType@MTEF@5@5@+=feaafiart1ev1aaatCvAUfKttLearuWrP9MDH5MBPbIqV92AaeXatLxBI9gBaebbnrfifHhDYfgasaacH8akY=wiFfYdH8Gipec8Eeeu0xXdbba9frFj0=OqFfea0dXdd9vqai=hGuQ8kuc9pgc9s8qqaq=dirpe0xb9q8qiLsFr0=vr0=vr0dc8meaabaqaciaacaGaaeqabaqabeGadaaakeaacqqHuoarcuWGybawgaacamaaBaaaleaacqGGOaakcqWGPbqAcqGGUaGlcqGGUaGlcqWGQbGAcqGGPaqkaeqaaOGaeyypa0ZaamWaaeaafaqabeqafaaaaeaacqqHuoarcuWG4baEgaacamaaBaaaleaacqWGPbqAaeqaaaGcbaGaeuiLdqKafmiEaGNbaGaadaWgaaWcbaGaemyAaKMaey4kaSIaeGymaedabeaaaOqaaiablAcilbqaaiabfs5aejqbdIha4zaaiaWaaSbaaSqaaiabdQgaQjabgkHiTiabigdaXaqabaaakeaacqqHuoarcuWG4baEgaacamaaBaaaleaacqWGQbGAaeqaaaaaaOGaay5waiaaw2faaiabcYcaSiaaxMaacaWLjaWaaeWaaeaacqaIYaGmcqaIWaamcqqGHbqyaiaawIcacaGLPaaaaaa@5674@

V(i..j)=[−vi−vi+1…−vj−1−vj]     (20b)
 MathType@MTEF@5@5@+=feaafiart1ev1aaatCvAUfKttLearuWrP9MDH5MBPbIqV92AaeXatLxBI9gBaebbnrfifHhDYfgasaacH8akY=wiFfYdH8Gipec8Eeeu0xXdbba9frFj0=OqFfea0dXdd9vqai=hGuQ8kuc9pgc9s8qqaq=dirpe0xb9q8qiLsFr0=vr0=vr0dc8meaabaqaciaacaGaaeqabaqabeGadaaakeaacqWGwbGvdaWgaaWcbaGaeiikaGIaemyAaKMaeiOla4IaeiOla4IaemOAaOMaeiykaKcabeaakiabg2da9maadmaabaqbaeqabeqbaaaabaGaeyOeI0IaemODay3aaSbaaSqaaiabdMgaPbqabaaakeaacqGHsislcqWG2bGDdaWgaaWcbaGaemyAaKMaey4kaSIaeGymaedabeaaaOqaaiablAcilbqaaiabgkHiTiabdAha2naaBaaaleaacqWGQbGAcqGHsislcqaIXaqmaeqaaaGcbaGaeyOeI0IaemODay3aaSbaaSqaaiabdQgaQbqabaaaaaGccaGLBbGaayzxaaGaaCzcaiaaxMaadaqadaqaaiabikdaYiabicdaWiabbkgaIbGaayjkaiaawMcaaaaa@51ED@

for *i *<*j *(*i *and *j *are positive integer numbers).

To formulate the problem in a standard least squares form, i.e. *Ax *= *b*, where *A *and *b *are measurements and *x *is to be estimated, we make the following definitions:

A:=[ΔX˜(1..(L−1))11×(L−1)]T∈ℝ(L−1)×(n+1),     (21a)
 MathType@MTEF@5@5@+=feaafiart1ev1aaatCvAUfKttLearuWrP9MDH5MBPbIqV92AaeXatLxBI9gBaebbnrfifHhDYfgasaacH8akY=wiFfYdH8Gipec8Eeeu0xXdbba9frFj0=OqFfea0dXdd9vqai=hGuQ8kuc9pgc9s8qqaq=dirpe0xb9q8qiLsFr0=vr0=vr0dc8meaabaqaciaacaGaaeqabaqabeGadaaakeaacqWGbbqqcqGG6aGocqGH9aqpdaWadaqaauaabeqaceaaaeaacqqHuoarcuWGybawgaacamaaBaaaleaacqGGOaakcqaIXaqmcqGGUaGlcqGGUaGlcqGGOaakcqWGmbatcqGHsislcqaIXaqmcqGGPaqkcqGGPaqkaeqaaaGcbaacbeGae8xmaeZaaSbaaSqaaiabigdaXiabgEna0kabcIcaOiabdYeamjabgkHiTiabigdaXiabcMcaPaqabaaaaaGccaGLBbGaayzxaaWaaWbaaSqabeaacqWGubavaaGccqGHiiIZcqWIDesOdaahaaWcbeqaaiabcIcaOiabdYeamjabgkHiTiabigdaXiabcMcaPiabgEna0kabcIcaOiabd6gaUjabgUcaRiabigdaXiabcMcaPaaakiabcYcaSiaaxMaacaWLjaWaaeWaaeaacqaIYaGmcqaIXaqmcqqGHbqyaiaawIcacaGLPaaaaaa@5DAF@

b:=[the i-th row of ΔX˜(2..L)]T∈ℝ(L−1)×1,     (21b)
 MathType@MTEF@5@5@+=feaafiart1ev1aaatCvAUfKttLearuWrP9MDH5MBPbIqV92AaeXatLxBI9gBaebbnrfifHhDYfgasaacH8akY=wiFfYdH8Gipec8Eeeu0xXdbba9frFj0=OqFfea0dXdd9vqai=hGuQ8kuc9pgc9s8qqaq=dirpe0xb9q8qiLsFr0=vr0=vr0dc8meaabaqaciaacaGaaeqabaqabeGadaaakeaacqWGIbGycqGG6aGocqGH9aqpdaWadaqaaiabbsha0jabbIgaOjabbwgaLjabbccaGiabdMgaPjabb2caTiabbsha0jabbIgaOjabbccaGiabbkhaYjabb+gaVjabbEha3jabbccaGiabb+gaVjabbAgaMjabbccaGiabfs5aejqbdIfayzaaiaWaaSbaaSqaaiabcIcaOiabikdaYiabc6caUiabc6caUiabdYeamjabcMcaPaqabaaakiaawUfacaGLDbaadaahaaWcbeqaaiabdsfaubaakiabgIGiolabl2riHoaaCaaaleqabaGaeiikaGIaemitaWKaeyOeI0IaeGymaeJaeiykaKIaey41aqRaeGymaedaaOGaeiilaWIaaCzcaiaaxMaadaqadaqaaiabikdaYiabigdaXiabbkgaIbGaayjkaiaawMcaaaaa@60B4@

x:={the i-th row of [Φ  u]}T∈ℝ(n×1)×1     (21c)
 MathType@MTEF@5@5@+=feaafiart1ev1aaatCvAUfKttLearuWrP9MDH5MBPbIqV92AaeXatLxBI9gBaebbnrfifHhDYfgasaacH8akY=wiFfYdH8Gipec8Eeeu0xXdbba9frFj0=OqFfea0dXdd9vqai=hGuQ8kuc9pgc9s8qqaq=dirpe0xb9q8qiLsFr0=vr0=vr0dc8meaabaqaciaacaGaaeqabaqabeGadaaakeaacqWG4baEcqGG6aGocqGH9aqpdaGadeqaaiabbsha0jabbIgaOjabbwgaLjabbccaGiabdMgaPjabb2caTiabbsha0jabbIgaOjabbccaGiabbkhaYjabb+gaVjabbEha3jabbccaGiabb+gaVjabbAgaMjabbccaGmaadmaabaGaeuOPdyKaeeiiaaIaeeiiaaIaemyDauhacaGLBbGaayzxaaaacaGL7bGaayzFaaWaaWbaaSqabeaacqWGubavaaGccqGHiiIZcqWIDesOdaahaaWcbeqaaiabcIcaOiabd6gaUjabgEna0kabigdaXiabcMcaPiabgEna0kabigdaXaaakiaaxMaacaWLjaWaaeWaaeaacqaIYaGmcqaIXaqmcqqGJbWyaiaawIcacaGLPaaaaaa@5FAC@

for *i *= 1, 2, ..., *n*. Now, the *i*-th row of the matrix [Φ *u*] in (19) can be written in the standard form as follows:

(*A *+ Δ*A*) *x *= *b *+ Δ*b *    (22)

where

ΔA:=[V(1..(L−1))01×(L−1)]T,     (23a)
 MathType@MTEF@5@5@+=feaafiart1ev1aaatCvAUfKttLearuWrP9MDH5MBPbIqV92AaeXatLxBI9gBaebbnrfifHhDYfgasaacH8akY=wiFfYdH8Gipec8Eeeu0xXdbba9frFj0=OqFfea0dXdd9vqai=hGuQ8kuc9pgc9s8qqaq=dirpe0xb9q8qiLsFr0=vr0=vr0dc8meaabaqaciaacaGaaeqabaqabeGadaaakeaacqqHuoarcqWGbbqqcqGG6aGocqGH9aqpdaWadaqaauaabeqaceaaaeaacqWGwbGvdaWgaaWcbaGaeiikaGIaeGymaeJaeiOla4IaeiOla4IaeiikaGIaemitaWKaeyOeI0IaeGymaeJaeiykaKIaeiykaKcabeaaaOqaaGqabiab=bdaWmaaBaaaleaacqaIXaqmcqGHxdaTcqGGOaakcqWGmbatcqGHsislcqaIXaqmcqGGPaqkaeqaaaaaaOGaay5waiaaw2faamaaCaaaleqabaGaemivaqfaaOGaeiilaWIaaCzcaiaaxMaadaqadaqaaiabikdaYiabiodaZiabbggaHbGaayjkaiaawMcaaaaa@4EC3@

Δb:=[the i-th row of V(2..L)]T     (23b)
 MathType@MTEF@5@5@+=feaafiart1ev1aaatCvAUfKttLearuWrP9MDH5MBPbIqV92AaeXatLxBI9gBaebbnrfifHhDYfgasaacH8akY=wiFfYdH8Gipec8Eeeu0xXdbba9frFj0=OqFfea0dXdd9vqai=hGuQ8kuc9pgc9s8qqaq=dirpe0xb9q8qiLsFr0=vr0=vr0dc8meaabaqaciaacaGaaeqabaqabeGadaaakeaacqqHuoarcqWGIbGycqGG6aGocqGH9aqpdaWadaqaaiabbsha0jabbIgaOjabbwgaLjabbccaGiabdMgaPjabb2caTiabbsha0jabbIgaOjabbccaGiabbkhaYjabb+gaVjabbEha3jabbccaGiabb+gaVjabbAgaMjabbccaGiabdAfawnaaBaaaleaacqGGOaakcqaIYaGmcqGGUaGlcqGGUaGlcqWGmbatcqGGPaqkaeqaaaGccaGLBbGaayzxaaWaaWbaaSqabeaacqWGubavaaGccaWLjaGaaCzcamaabmaabaGaeGOmaiJaeG4mamJaeeOyaigacaGLOaGaayzkaaaaaa@54E3@

for *i *= 1, 2, ..., *n*. Δ*A *and Δ*b *are unknown correction terms caused by the noise in the data. The above problem is solved *n*-times to obtain the estimate of all the rows in the matrix Φ. For the case of multiple experiments, the details of the formulation of the problem are given in the additional file (See Additional File 1).

### Estimating the Jacobian in the Presence of Noise

Consider first the simple scalar version of the least squares problem in the absence of measurement noise, given by a* *x *= b*. In this case it is easy to see that the exact solution is given by x* = b*/a* for a* ≠ 0. Now, let the measurements of a* and b* be corrupted by noise as follows: *a *= a* + *v*_1 _and *b *= b* + *v*_2 _where a* and b* are the true values, *v*_1 _and *v*_2 _are the unknown measurement noise, and *a *and *b *are the (known) measurements of a* and b*. In this case, the standard least squares solution for just one set of measurements is

xLS=ba=b∗+v2a∗+v1=(b∗/a∗)+(v2/a∗)1+(v1/a∗).     (24)
 MathType@MTEF@5@5@+=feaafiart1ev1aaatCvAUfKttLearuWrP9MDH5MBPbIqV92AaeXatLxBI9gBaebbnrfifHhDYfgasaacH8akY=wiFfYdH8Gipec8Eeeu0xXdbba9frFj0=OqFfea0dXdd9vqai=hGuQ8kuc9pgc9s8qqaq=dirpe0xb9q8qiLsFr0=vr0=vr0dc8meaabaqaciaacaGaaeqabaqabeGadaaakeaacqWG4baEdaWgaaWcbaGaeeitaWKaee4uamfabeaakiabg2da9maalaaabaGaemOyaigabaGaemyyaegaaiabg2da9maalaaabaGaemOyai2aaWbaaSqabeaacqGHxiIkaaGccqGHRaWkcqWG2bGDdaWgaaWcbaGaeGOmaidabeaaaOqaaiabdggaHnaaCaaaleqabaGaey4fIOcaaOGaey4kaSIaemODay3aaSbaaSqaaiabigdaXaqabaaaaOGaeyypa0ZaaSaaaeaacqGGOaakcqWGIbGydaahaaWcbeqaaiabgEHiQaaakiabc+caViabdggaHnaaCaaaleqabaGaey4fIOcaaOGaeiykaKIaey4kaSIaeiikaGIaemODay3aaSbaaSqaaiabikdaYaqabaGccqGGVaWlcqWGHbqydaahaaWcbeqaaiabgEHiQaaakiabcMcaPaqaaiabigdaXiabgUcaRiabcIcaOiabdAha2naaBaaaleaacqaIXaqmaeqaaOGaei4la8Iaemyyae2aaWbaaSqabeaacqGHxiIkaaGccqGGPaqkaaGaeiOla4IaaCzcaiaaxMaadaqadaqaaiabikdaYiabisda0aGaayjkaiaawMcaaaaa@6174@

Using the binomial theorem, the denominator of the above expression can be expanded as follows:

11+(v1/a∗)=1−v1a∗+(v1a∗)2−(v1a∗)3+…  for|v1a∗|<1.     (25)
 MathType@MTEF@5@5@+=feaafiart1ev1aaatCvAUfKttLearuWrP9MDH5MBPbIqV92AaeXatLxBI9gBaebbnrfifHhDYfgasaacH8akY=wiFfYdH8Gipec8Eeeu0xXdbba9frFj0=OqFfea0dXdd9vqai=hGuQ8kuc9pgc9s8qqaq=dirpe0xb9q8qiLsFr0=vr0=vr0dc8meaabaqaciaacaGaaeqabaqabeGadaaakeaadaWcaaqaaiabigdaXaqaaiabigdaXiabgUcaRiabcIcaOiabdAha2naaBaaaleaacqaIXaqmaeqaaOGaei4la8Iaemyyae2aaWbaaSqabeaacqGHxiIkaaGccqGGPaqkaaGaeyypa0JaeGymaeJaeyOeI0YaaSaaaeaacqWG2bGDdaWgaaWcbaGaeGymaedabeaaaOqaaiabdggaHnaaCaaaleqabaGaey4fIOcaaaaakiabgUcaRmaabmaabaWaaSaaaeaacqWG2bGDdaWgaaWcbaGaeGymaedabeaaaOqaaiabdggaHnaaCaaaleqabaGaey4fIOcaaaaaaOGaayjkaiaawMcaamaaCaaaleqabaGaeGOmaidaaOGaeyOeI0YaaeWaaeaadaWcaaqaaiabdAha2naaBaaaleaacqaIXaqmaeqaaaGcbaGaemyyae2aaWbaaSqabeaacqGHxiIkaaaaaaGccaGLOaGaayzkaaWaaWbaaSqabeaacqaIZaWmaaGccqGHRaWkcqWIMaYscqqGGaaicqqGGaaicqqGMbGzcqqGVbWBcqqGYbGCdaabdaqaamaalaaabaGaemODay3aaSbaaSqaaiabigdaXaqabaaakeaacqWGHbqydaahaaWcbeqaaiabgEHiQaaaaaaakiaawEa7caGLiWoacqGH8aapcqaIXaqmcqGGUaGlcaWLjaGaaCzcamaabmaabaGaeGOmaiJaeGynaudacaGLOaGaayzkaaaaaa@6810@

Then, the least squares solution becomes

xLS=b∗a∗[1−v1a∗+(v1a∗)2−(v1a∗)3+…]+v2a∗[1−v1a∗+(v1a∗)2−(v1a∗)3+…].     (26)
 MathType@MTEF@5@5@+=feaafiart1ev1aaatCvAUfKttLearuWrP9MDH5MBPbIqV92AaeXatLxBI9gBaebbnrfifHhDYfgasaacH8akY=wiFfYdH8Gipec8Eeeu0xXdbba9frFj0=OqFfea0dXdd9vqai=hGuQ8kuc9pgc9s8qqaq=dirpe0xb9q8qiLsFr0=vr0=vr0dc8meaabaqaciaacaGaaeqabaqabeGadaaakeaacqWG4baEdaWgaaWcbaGaeeitaWKaee4uamfabeaakiabg2da9maalaaabaGaemOyai2aaWbaaSqabeaacqGHxiIkaaaakeaacqWGHbqydaahaaWcbeqaaiabgEHiQaaaaaGcdaWadaqaaiabigdaXiabgkHiTmaalaaabaGaemODay3aaSbaaSqaaiabigdaXaqabaaakeaacqWGHbqydaahaaWcbeqaaiabgEHiQaaaaaGccqGHRaWkdaqadaqaamaalaaabaGaemODay3aaSbaaSqaaiabigdaXaqabaaakeaacqWGHbqydaahaaWcbeqaaiabgEHiQaaaaaaakiaawIcacaGLPaaadaahaaWcbeqaaiabikdaYaaakiabgkHiTmaabmaabaWaaSaaaeaacqWG2bGDdaWgaaWcbaGaeGymaedabeaaaOqaaiabdggaHnaaCaaaleqabaGaey4fIOcaaaaaaOGaayjkaiaawMcaamaaCaaaleqabaGaeG4mamdaaOGaey4kaSIaeSOjGSeacaGLBbGaayzxaaGaey4kaSYaaSaaaeaacqWG2bGDdaWgaaWcbaGaeGOmaidabeaaaOqaaiabdggaHnaaCaaaleqabaGaey4fIOcaaaaakmaadmaabaGaeGymaeJaeyOeI0YaaSaaaeaacqWG2bGDdaWgaaWcbaGaeGymaedabeaaaOqaaiabdggaHnaaCaaaleqabaGaey4fIOcaaaaakiabgUcaRmaabmaabaWaaSaaaeaacqWG2bGDdaWgaaWcbaGaeGymaedabeaaaOqaaiabdggaHnaaCaaaleqabaGaey4fIOcaaaaaaOGaayjkaiaawMcaamaaCaaaleqabaGaeGOmaidaaOGaeyOeI0YaaeWaaeaadaWcaaqaaiabdAha2naaBaaaleaacqaIXaqmaeqaaaGcbaGaemyyae2aaWbaaSqabeaacqGHxiIkaaaaaaGccaGLOaGaayzkaaWaaWbaaSqabeaacqaIZaWmaaGccqGHRaWkcqWIMaYsaiaawUfacaGLDbaacqGGUaGlcaWLjaGaaCzcamaabmaabaGaeGOmaiJaeGOnaydacaGLOaGaayzkaaaaaa@7AF6@

Now, if both *v*_1 _and *v*_2 _are independent zero-mean white gaussian noises, this implies that E(*v*_1_) = 0, E(*v*_2_) = 0, E(*v*_1 _*v*_2_) = 0, *etc*. Thus, taking the Expectation on both sides gives

E(xLS)≈b∗a∗+b∗a∗3σ12=x∗+b∗a∗3σ12     (27)
 MathType@MTEF@5@5@+=feaafiart1ev1aaatCvAUfKttLearuWrP9MDH5MBPbIqV92AaeXatLxBI9gBaebbnrfifHhDYfgasaacH8akY=wiFfYdH8Gipec8Eeeu0xXdbba9frFj0=OqFfea0dXdd9vqai=hGuQ8kuc9pgc9s8qqaq=dirpe0xb9q8qiLsFr0=vr0=vr0dc8meaabaqaciaacaGaaeqabaqabeGadaaakeaacqqGfbqrcqGGOaakcqWG4baEdaWgaaWcbaGaeeitaWKaee4uamfabeaakiabcMcaPiabgIKi7oaalaaabaGaemOyai2aaWbaaSqabeaacqGHxiIkaaaakeaacqWGHbqydaahaaWcbeqaaiabgEHiQaaaaaGccqGHRaWkdaWcaaqaaiabdkgaInaaCaaaleqabaGaey4fIOcaaaGcbaGaemyyae2aaWbaaSqabeaacqGHxiIkcqaIZaWmaaaaaGGacOGae83Wdm3aa0baaSqaaiabigdaXaqaaiabikdaYaaakiabg2da9iabdIha4naaCaaaleqabaGaey4fIOcaaOGaey4kaSYaaSaaaeaacqWGIbGydaahaaWcbeqaaiabgEHiQaaaaOqaaiabdggaHnaaCaaaleqabaGaey4fIOIaeG4mamdaaaaakiab=n8aZnaaDaaaleaacqaIXaqmaeaacqaIYaGmaaGccaWLjaGaaCzcamaabmaabaGaeGOmaiJaeG4naCdacaGLOaGaayzkaaaaaa@57BD@

where σ12
 MathType@MTEF@5@5@+=feaafiart1ev1aaatCvAUfKttLearuWrP9MDH5MBPbIqV92AaeXatLxBI9gBaebbnrfifHhDYfgasaacH8akY=wiFfYdH8Gipec8Eeeu0xXdbba9frFj0=OqFfea0dXdd9vqai=hGuQ8kuc9pgc9s8qqaq=dirpe0xb9q8qiLsFr0=vr0=vr0dc8meaabaqaciaacaGaaeqabaqabeGadaaakeaaiiGacqWFdpWCdaqhaaWcbaGaeGymaedabaGaeGOmaidaaaaa@3085@ is the variance of *v*_1_. It is clear from the above relation that if the noise *v*_1 _is not present, the least squares solution gives the true solution in the average sense. However, when a* is corrupted by noise, the solution is not optimal but biased proportional to the variance of the noise. To correct this situation, the problem is now set up with so-called correction terms as follows:

(*a *+ Δ*a*) *x *= *b *+ Δ*b*.     (28)

The Total Least Squares (TLS) technique was developed to solve exactly this problem by finding the correction terms Δ*a *and Δ*b*. The correction terms are obtained by minimising ||Δ*a *Δ*b*||_F_, while simultaneously satisfying the above relation (28) where || · ||_*F *_is the Frobenius norm defined by ||*A*||_*F *_= tr(AAT)
 MathType@MTEF@5@5@+=feaafiart1ev1aaatCvAUfKttLearuWrP9MDH5MBPbIqV92AaeXatLxBI9gBaebbnrfifHhDYfgasaacH8akY=wiFfYdH8Gipec8Eeeu0xXdbba9frFj0=OqFfea0dXdd9vqai=hGuQ8kuc9pgc9s8qqaq=dirpe0xb9q8qiLsFr0=vr0=vr0dc8meaabaqaciaacaGaaeqabaqabeGadaaakeaadaGcaaqaaiabbsha0jabbkhaYjabcIcaOiabdgeabjabdgeabnaaCaaaleqabaGaemivaqfaaOGaeiykaKcaleqaaaaa@34D1@ for a matrix *A *in which tr(*AA*^*T*^) is the trace of the matrix, i.e. the sum of the diagonal terms. For the case of one measurement, as given above, the cost minimised by the TLS is given by

||Δ*a *Δ*b*||_*F *_= Δ*a*^2 ^+ Δ*b*^2^.     (29)

For a higher number of measurements, i.e. *Ax *= *b*, the solution from the TLS is given by

*x*_TLS _= (*A*^*T *^*A *- *λ*^2 ^*I*)^-1 ^*A*^T ^*b *    (30)

where *λ *is the smallest singular value of [*A b*] and the derivation can be found in the additional file (See Additional File 1) or [7]. On the other hand, the conventional least squares solution is given as follows:

*x*_LS _= (*A*^T ^*A*)^-1 ^*A*^*T *^*b *    (31)

and thus it essentially has the same error as shown in (27). The TLS technique tries to find the correction terms for *A *such that the bias error, which stems from the inaccuracy in *A*, is reduced. Hence, the quality of *x*_TLS _depends on how close the estimated *λ *is to the true correction term. The TLS solution is always guaranteed to be as good or better than the least squares solution in the root mean square sense, if the number of measurements is sufficient to allow the algorithm to compute a reasonable approximation of the true correction term. If the number of measurements is too small, however, the TLS solution may not be better than the conventional least squares solution.

One of the main assumptions behind the TLS technique is that the two noise terms *v*_1 _and *v*_2 _are independent. However, if they are not independent but related to each other in some way, this knowledge about the structure of the problem should be used in estimating the solution. For example, if *v*_1 _= *v*_2_, the least squares solution is approximated by

E(xLS)≈x∗+b∗a∗3σ12−1a∗2σ12.     (32)
 MathType@MTEF@5@5@+=feaafiart1ev1aaatCvAUfKttLearuWrP9MDH5MBPbIqV92AaeXatLxBI9gBaebbnrfifHhDYfgasaacH8akY=wiFfYdH8Gipec8Eeeu0xXdbba9frFj0=OqFfea0dXdd9vqai=hGuQ8kuc9pgc9s8qqaq=dirpe0xb9q8qiLsFr0=vr0=vr0dc8meaabaqaciaacaGaaeqabaqabeGadaaakeaacqqGfbqrcqGGOaakcqWG4baEdaWgaaWcbaGaeeitaWKaee4uamfabeaakiabcMcaPiabgIKi7kabdIha4naaCaaaleqabaGaey4fIOcaaOGaey4kaSYaaSaaaeaacqWGIbGydaahaaWcbeqaaiabgEHiQaaaaOqaaiabdggaHnaaCaaaleqabaGaey4fIOIaeG4mamdaaaaaiiGakiab=n8aZnaaDaaaleaacqaIXaqmaeaacqaIYaGmaaGccqGHsisldaWcaaqaaiabigdaXaqaaiabdggaHnaaCaaaleqabaGaey4fIOIaeGOmaidaaaaakiab=n8aZnaaDaaaleaacqaIXaqmaeaacqaIYaGmaaGccqGGUaGlcaWLjaGaaCzcamaabmaabaGaeG4mamJaeGOmaidacaGLOaGaayzkaaaaaa@5125@

The optimal solution for this case is the Constrained Total Least Squares (CTLS) technique. If it is known that *v*_1 _= *v*_2 _then Δ*a *must be equal to Δ*b*. Hence, instead of minimising the Frobenius norm of ||Δ*a *Δ*b*||_F_, a more appropriate cost for this problem would be Δ*a*^2 ^instead of Δ*a*^2 ^+ Δ*b*^2^. The CTLS algorithm exploits the knowledge that the true correction terms must be of the form Δ*a *= -*v*_1 _and Δ*b *= -*v*_1_. As a result, the CTLS technique searches for the correction term, which is a minimum in the 2-norm sense, i.e. ‖v1‖22
 MathType@MTEF@5@5@+=feaafiart1ev1aaatCvAUfKttLearuWrP9MDH5MBPbIqV92AaeXatLxBI9gBaebbnrfifHhDYfgasaacH8akY=wiFfYdH8Gipec8Eeeu0xXdbba9frFj0=OqFfea0dXdd9vqai=hGuQ8kuc9pgc9s8qqaq=dirpe0xb9q8qiLsFr0=vr0=vr0dc8meaabaqaciaacaGaaeqabaqabeGadaaakeaadaqbdaqaaiabdAha2naaBaaaleaacqaIXaqmaeqaaaGccaGLjWUaayPcSdWaa0baaSqaaiabikdaYaqaaiabikdaYaaaaaa@347F@, and simultaneously satisfies the constraint (28) where ‖v1‖22=v1Tv1
 MathType@MTEF@5@5@+=feaafiart1ev1aaatCvAUfKttLearuWrP9MDH5MBPbIqV92AaeXatLxBI9gBaebbnrfifHhDYfgasaacH8akY=wiFfYdH8Gipec8Eeeu0xXdbba9frFj0=OqFfea0dXdd9vqai=hGuQ8kuc9pgc9s8qqaq=dirpe0xb9q8qiLsFr0=vr0=vr0dc8meaabaqaciaacaGaaeqabaqabeGadaaakeaadaqbdaqaaiabdAha2naaBaaaleaacqaIXaqmaeqaaaGccaGLjWUaayPcSdWaa0baaSqaaiabikdaYaqaaiabikdaYaaakiabg2da9iabdAha2naaDaaaleaacqaIXaqmaeaacqWGubavaaGccqWG2bGDdaWgaaWcbaGaeGymaedabeaaaaa@3BED@. Other than different cost functions for each method, the main difference between the TLS and the CTLS is the dimension of the correction term search space. For one set of measurements, for example, the TLS searches for the correction terms Δ*a *and Δ*b *in a two dimensional space, while the CTLS, on the other hand, searches for the single correction term Δ*a *or Δ*b *in the minimal (one dimensional) space. The CTLS formulation can finally be reduced to the following minimisation problem:

min⁡x[xT−1]CT(HxHxT)−1C[x−1]     (33)
 MathType@MTEF@5@5@+=feaafiart1ev1aaatCvAUfKttLearuWrP9MDH5MBPbIqV92AaeXatLxBI9gBaebbnrfifHhDYfgasaacH8akY=wiFfYdH8Gipec8Eeeu0xXdbba9frFj0=OqFfea0dXdd9vqai=hGuQ8kuc9pgc9s8qqaq=dirpe0xb9q8qiLsFr0=vr0=vr0dc8meaabaqaciaacaGaaeqabaqabeGadaaakeaadaWfqaqaaiGbc2gaTjabcMgaPjabc6gaUbWcbaGaemiEaGhabeaakmaadmaabaqbaeqabeGaaaqaaiabdIha4naaCaaaleqabaGaemivaqfaaaGcbaGaeyOeI0IaeGymaedaaaGaay5waiaaw2faaiabdoeadnaaCaaaleqabaGaemivaqfaaOWaaeWaaeaacqWGibasdaWgaaWcbaGaemiEaGhabeaakiabdIeainaaDaaaleaacqWG4baEaeaacqWGubavaaaakiaawIcacaGLPaaadaahaaWcbeqaaiabgkHiTiabigdaXaaakiabdoeadnaadmaabaqbaeqabiqaaaqaaiabdIha4bqaaiabgkHiTiabigdaXaaaaiaawUfacaGLDbaacaWLjaGaaCzcamaabmaabaGaeG4mamJaeG4mamdacaGLOaGaayzkaaaaaa@5136@

where *C *is constructed from the measurements and *H*_*x *_is given in a special form which is a function of the structure of the correction terms and also of *x *– the details can be found in the additional file (See Additional File 1) or in [10]. The minimisation problem solved by the CTLS algorithm is nonlinear since *H*_*x *_is a function of *x *and in general will not be convex – therefore the quality of the resulting solution will depend on the initial guess for *x*. Of course the simplest way to obtain a good initial guess is to use the solution provided by the LS algorithm. If this solution is not too far away from the true solution, then the minimisation problem can be efficiently solved by some local minimisation algorithm such as Newton's method, Sequential Quadratic Programming, *etc *[16]. However, in some cases it may happen that the LS solution does not provide a good initial guess and, as a result, the minimisation algorithm may produce very large values for Δ*a *and Δ*b*. In general, large magnitudes of the correction terms correspond to incorrect solutions because if Δ*a *and Δ*b *are large compared to the magnitude of *a *and *b*, then Δ*a *and Δ*b *become dominant and the solution could be any number. For example, if a* and b* are equal to 1 and the measurements, i.e. *a *and *b*, are 1.1 and 0.9, respectively, the correct correction terms are -0.1 and 0.1. However, if the correction terms are given as 1 × 10^10 ^and -1 × 10^100^, then the solution is approximately -1 × 10^90^, which is too far from the true solution, 1. Hence, whenever the final solution produced by the CTLS algorithm is drastically different from the initial guess in terms of the magnitude, it is advisable to impose some bounds on the magnitude of the correction terms. To do this, the above unconstrained minimisation problem may be solved with the following constraint on *x*:

*x*_0 _- *h *|*x*_0_| ≤ *x *≤ *x*_0 _+ *h *|*x*_0_|     (34)

where *h *is a small positive constant. Numerically, constrained optimisation problems are much harder to solve than unconstrained optimisation problems, and hence, the original unconstrained problem should generally be solved first, with the above constraints only being imposed if they are necessary to compute a reasonable solution.

Finally, we note that there are many cases of biochemical experimental data where the matrix inversions required in (31) and (33) are not possible, i.e., they are singular or very close to singular. This is usually a result of a too large sampling time which results in the *A *matrix not having full-rank. One way to fix this situation is by removing the dependent parts of *A *by using a singular value decomposition. After eliminating the dependent parts, the matrix inversions in (31) and (33) are feasible and the problems are again well-defined. More details can be found in [1]. Full details of the mathematics involved in the solutions of the TLS and CTLS problems are given in the additional files (See Additional File 1), together with complete MATLAB programmes for the solution of each algorithm (See Additional File 2).

### Evaluating the Jacobian Estimation Error

The main reason for estimating the Jacobian is to gain a quantitative understanding of the local structure of the biochemical network. Hence, correct estimation of each element of the matrix is important, since each element provides a measure of the (local) functional interaction between two nodes in the network. From this point of view, the estimation error, *ε*_*M*_, can be naturally defined as follows:

εM:=1N1∑i=1n∑j=1n|αij|+1N2∑i=1n∑j=1n|βij|     (35)
 MathType@MTEF@5@5@+=feaafiart1ev1aaatCvAUfKttLearuWrP9MDH5MBPbIqV92AaeXatLxBI9gBaebbnrfifHhDYfgasaacH8akY=wiFfYdH8Gipec8Eeeu0xXdbba9frFj0=OqFfea0dXdd9vqai=hGuQ8kuc9pgc9s8qqaq=dirpe0xb9q8qiLsFr0=vr0=vr0dc8meaabaqaciaacaGaaeqabaqabeGadaaakeaaiiGacqWF1oqzdaWgaaWcbaGaemyta0eabeaakiabcQda6iabg2da9maalaaabaGaeGymaedabaGaemOta40aaSbaaSqaaiabigdaXaqabaaaaOWaaabCaeaadaaeWbqaamaaemaabaGae8xSde2aaSbaaSqaaiabdMgaPjabdQgaQbqabaaakiaawEa7caGLiWoaaSqaaiabdQgaQjabg2da9iabigdaXaqaaiabd6gaUbqdcqGHris5aaWcbaGaemyAaKMaeyypa0JaeGymaedabaGaemOBa4ganiabggHiLdGccqGHRaWkdaWcaaqaaiabigdaXaqaaiabd6eaonaaBaaaleaacqaIYaGmaeqaaaaakmaaqahabaWaaabCaeaadaabdaqaaiab=j7aInaaBaaaleaacqWGPbqAcqWGQbGAaeqaaaGccaGLhWUaayjcSdaaleaacqWGQbGAcqGH9aqpcqaIXaqmaeaacqWGUbGBa0GaeyyeIuoaaSqaaiabdMgaPjabg2da9iabigdaXaqaaiabd6gaUbqdcqGHris5aOGaaCzcaiaaxMaadaqadaqaaiabiodaZiabiwda1aGaayjkaiaawMcaaaaa@6936@

where *N*_1 _is the number of non-zero elements in the true *F*, *N*_2 _is the number of zero elements in the true *F*, *F *is given by

*F *:= (*f*_*ij*_)_*n *× *n*_,     (36)

αij:={f^ij−fijfij,for fij≠00,otherwise      (37a)
 MathType@MTEF@5@5@+=feaafiart1ev1aaatCvAUfKttLearuWrP9MDH5MBPbIqV92AaeXatLxBI9gBaebbnrfifHhDYfgasaacH8akY=wiFfYdH8Gipec8Eeeu0xXdbba9frFj0=OqFfea0dXdd9vqai=hGuQ8kuc9pgc9s8qqaq=dirpe0xb9q8qiLsFr0=vr0=vr0dc8meaabaqaciaacaGaaeqabaqabeGadaaakeaaiiGacqWFXoqydaWgaaWcbaGaemyAaKMaemOAaOgabeaakiabcQda6iabg2da9maaceqabaqbaeaabiGaaaqaamaalaaabaGafmOzayMbaKaadaWgaaWcbaGaemyAaKMaemOAaOgabeaakiabgkHiTiabdAgaMnaaBaaaleaacqWGPbqAcqWGQbGAaeqaaaGcbaGaemOzay2aaSbaaSqaaiabdMgaPjabdQgaQbqabaaaaOGaeiilaWcabaGaeeOzayMaee4Ba8MaeeOCaiNaeeiiaaIaemOzay2aaSbaaSqaaiabdMgaPjabdQgaQbqabaGccqGHGjsUcqaIWaamaeaacqaIWaamcqGGSaalaeaacqqGVbWBcqqG0baDcqqGObaAcqqGLbqzcqqGYbGCcqqG3bWDcqqGPbqAcqqGZbWCcqqGLbqzaaGaeeiiaacacaGL7baacaWLjaGaaCzcamaabmaabaGaeG4mamJaeG4naCJaeeyyaegacaGLOaGaayzkaaaaaa@6412@

βij:={0,for fij≠0f^ij,otherwise      (37b)
 MathType@MTEF@5@5@+=feaafiart1ev1aaatCvAUfKttLearuWrP9MDH5MBPbIqV92AaeXatLxBI9gBaebbnrfifHhDYfgasaacH8akY=wiFfYdH8Gipec8Eeeu0xXdbba9frFj0=OqFfea0dXdd9vqai=hGuQ8kuc9pgc9s8qqaq=dirpe0xb9q8qiLsFr0=vr0=vr0dc8meaabaqaciaacaGaaeqabaqabeGadaaakeaaiiGacqWFYoGydaWgaaWcbaGaemyAaKMaemOAaOgabeaakiabcQda6iabg2da9maaceqabaqbaeaabiGaaaqaaiabicdaWiabcYcaSaqaaiabbAgaMjabb+gaVjabbkhaYjabbccaGiabdAgaMnaaBaaaleaacqWGPbqAcqWGQbGAaeqaaOGaeyiyIKRaeGimaadabaGafmOzayMbaKaadaWgaaWcbaGaemyAaKMaemOAaOgabeaakiabcYcaSaqaaiabb+gaVjabbsha0jabbIgaOjabbwgaLjabbkhaYjabbEha3jabbMgaPjabbohaZjabbwgaLbaacqqGGaaiaiaawUhaaiaaxMaacaWLjaWaaeWaaeaacqaIZaWmcqaI3aWncqqGIbGyaiaawIcacaGLPaaaaaa@5A93@

where f^
 MathType@MTEF@5@5@+=feaafiart1ev1aaatCvAUfKttLearuWrP9MDH5MBPbIqV92AaeXatLxBI9gBaebbnrfifHhDYfgasaacH8akY=wiFfYdH8Gipec8Eeeu0xXdbba9frFj0=OqFfea0dXdd9vqai=hGuQ8kuc9pgc9s8qqaq=dirpe0xb9q8qiLsFr0=vr0=vr0dc8meaabaqaciaacaGaaeqabaqabeGadaaakeaacuWGMbGzgaqcaaaa@2E11@_*ij *_and *f*_*ij *_are the *i*-th row, *j*-th column element of F^
 MathType@MTEF@5@5@+=feaafiart1ev1aaatCvAUfKttLearuWrP9MDH5MBPbIqV92AaeXatLxBI9gBaebbnrfifHhDYfgasaacH8akY=wiFfYdH8Gipec8Eeeu0xXdbba9frFj0=OqFfea0dXdd9vqai=hGuQ8kuc9pgc9s8qqaq=dirpe0xb9q8qiLsFr0=vr0=vr0dc8meaabaqaciaacaGaaeqabaqabeGadaaakeaacuWGgbGrgaqcaaaa@2DD1@ and *F*, respectively, and F^
 MathType@MTEF@5@5@+=feaafiart1ev1aaatCvAUfKttLearuWrP9MDH5MBPbIqV92AaeXatLxBI9gBaebbnrfifHhDYfgasaacH8akY=wiFfYdH8Gipec8Eeeu0xXdbba9frFj0=OqFfea0dXdd9vqai=hGuQ8kuc9pgc9s8qqaq=dirpe0xb9q8qiLsFr0=vr0=vr0dc8meaabaqaciaacaGaaeqabaqabeGadaaakeaacuWGgbGrgaqcaaaa@2DD1@ is the estimated matrix whose form is the same as *F*. The above definition is a slight modification of the one proposed in [1] where *β*_*ij *_= 0 for all *i *= 1, 2, ..., *n *- 1, *n *was made and *j *= 1, 2, ..., *n *- 1, *n *(i.e. it effectively ignores errors in the estimation of the zero elements of the Jacobian).

In [5], two alternative measures of the error in the Jacobian estimation, *r*_*z *_and *r*_*nz*_, are defined. These measures quantify the error in the zero elements and non-zero elements of the estimated matrix, respectively. To do this, the elements of the estimated Jacobian are sorted according to their absolute values. For a given positive integer *n*_*h*_, the smallest *n*_*h *_elements of the estimated Jacobian are then set to zero. *r*_*z *_is then defined as the ratio of the number of zero elements in the estimated Jacobian to the number of zero elements in the true Jacobian. *r*_*nz *_is defined as the ratio of the number of non-zero elements in the estimated Jacobian whose signs are the same as the ones in the true Jacobian, to the number of non-zero elements in the true Jacobian. In this paper, we consider a slightly more compact version of this measure and define an error measure *ε*_*S *_as follows:

εS:=1n2∑i=1n∑j=1n|sign(f^ij)−sign(fij)|     (38)
 MathType@MTEF@5@5@+=feaafiart1ev1aaatCvAUfKttLearuWrP9MDH5MBPbIqV92AaeXatLxBI9gBaebbnrfifHhDYfgasaacH8akY=wiFfYdH8Gipec8Eeeu0xXdbba9frFj0=OqFfea0dXdd9vqai=hGuQ8kuc9pgc9s8qqaq=dirpe0xb9q8qiLsFr0=vr0=vr0dc8meaabaqaciaacaGaaeqabaqabeGadaaakeaaiiGacqWF1oqzdaWgaaWcbaGaem4uamfabeaakiabcQda6iabg2da9maalaaabaGaeGymaedabaGaemOBa42aaWbaaSqabeaacqaIYaGmaaaaaOWaaabCaeaadaaeWbqaamaaemaabaGaee4CamNaeeyAaKMaee4zaCMaeeOBa42aaeWaaeaacuWGMbGzgaqcamaaBaaaleaacqWGPbqAcqWGQbGAaeqaaaGccaGLOaGaayzkaaGaeyOeI0Iaee4CamNaeeyAaKMaee4zaCMaeeOBa42aaeWaaeaacqWGMbGzdaWgaaWcbaGaemyAaKMaemOAaOgabeaaaOGaayjkaiaawMcaaaGaay5bSlaawIa7aaWcbaGaemOAaOMaeyypa0JaeGymaedabaGaemOBa4ganiabggHiLdaaleaacqWGPbqAcqGH9aqpcqaIXaqmaeaacqWGUbGBa0GaeyyeIuoakiaaxMaacaWLjaWaaeWaaeaacqaIZaWmcqaI4aaoaiaawIcacaGLPaaaaaa@62BD@

where sign(*a*) is a function that has the sign of *a *as its value, i.e., -1, 0, 1, for *a *< 0, *a *= 0, and *a *> 0, respectively. Finally, the third possible error definition is based on the Frobenius norm of a matrix:

*ε*_*F *_:= ||F^
 MathType@MTEF@5@5@+=feaafiart1ev1aaatCvAUfKttLearuWrP9MDH5MBPbIqV92AaeXatLxBI9gBaebbnrfifHhDYfgasaacH8akY=wiFfYdH8Gipec8Eeeu0xXdbba9frFj0=OqFfea0dXdd9vqai=hGuQ8kuc9pgc9s8qqaq=dirpe0xb9q8qiLsFr0=vr0=vr0dc8meaabaqaciaacaGaaeqabaqabeGadaaakeaacuWGgbGrgaqcaaaa@2DD1@ - *F*||_*F*_.     (39)

This error measure arises naturally in the context of the TLS problem since this approach exactly minimises the Frobenius norm of the correction terms arising from the noise in the data, Δ*A *and Δ*b*.

## Authors' contributions

JK derived the mathematical details, implemented and tested the algorithms under the supervision of DGB and IP. DGB, IP, and KHC checked the mathematical derivations. JK and DGB wrote the first draft of the manuscript. PHH and KHC provided biological interpretations of the results and all authors contributed to the final manuscript.

## Supplementary Material

Additional file 1 Detailed mathematical descriptions of the least squares, total least squares, and constrained total least squares algorithms, for the multiple experiments case, are provided in this file. Click here for file

Additional file 2 This is a standard zip compressed file. It can be uncompressed using freely available software, such as winzip. In unix, it can be uncompressed using the *unzip *command. This file includes the MATLAB source files to run all the calculation for the examples in this paper. To run the files, the MATLAB and Optimization toolboxes for MATLAB are required. More details about each file can be found in "readme.txt". Click here for file
