# Supplementary material for: Least-squares methods for identifying biochemical regulatory networks from noisy measurements
Source: BMC Bioinformatics. 2007 Jan 10;8:8. doi: 10.1186/1471-2105-8-8 (PMC1793997; doi:10.1186/1471-2105-8-8)
Supplement: Additional file 1 — Detailed mathematical descriptions of the least squares, total least squares, and constrained total least squares algorithms, for the multiple experiments case, are provided in this file. [file 1471-2105-8-8-S1.pdf]

Least-squares methods for identifying biochemical regulatory networks from noisy measurements

Additional File #1:

The least-Squares solution for multiple experiments case

Jongrae Kim, Declan G. Bates, Ian Postlethwaite, Pet Heslop-Harrison, and Kwang-Hyun Cho

Three least squares algorithms, the conventional least squares (LS), the total least squares (TLS), and the constrained total least squares (CTLS), for multiple experiments case, are presented, which can be formulated as follows:

$$(A + \Delta A)\, x = b + \Delta b. \tag{A1}$$

I. Constructing $Ax = b$ for the multiple experiment case

If p experiments are performed around the same equilibrium point, then A and b are constructed as follows:

$$A = \begin{bmatrix} \Delta\tilde{X}^1_{(1..(L-1))} & \Delta\tilde{X}^2_{(1..(L-1))} & \dots & \Delta\tilde{X}^{p-1}_{(1..(L-1))} & \Delta\tilde{X}^{p}_{(1..(L-1))} \\ & & I_p \otimes \mathbf{1}_{1\times(L-1)} & & \end{bmatrix}^T, \tag{A2a}$$

$$b = \left\{ \text{the } i\text{-th row of } \begin{bmatrix} \Delta\tilde{X}^1_{(2..L)} & \Delta\tilde{X}^2_{(2..L)} & \dots & \Delta\tilde{X}^{p-1}_{(2..L)} & \Delta\tilde{X}^{p}_{(2..L)} \end{bmatrix} \right\}^T \tag{A2b}$$

where $\Delta\tilde{X}^k_{(i..j)}$ is the k-th set of experimental data for $k = 1,\, 2,\, \dots,\, p-1,\, p$, I_p is the $p \times p$ identity matrix and the Kronecker product, $\otimes$, for two matrices M and N is defined as

$$M \otimes N := \begin{bmatrix} M_{1,1}N & M_{1,2}N & \dots & M_{1,m-1}N & M_{1,m}N \\ M_{2,1}N & M_{2,2}N & \dots & M_{2,m-1}N & M_{2,m}N \\ \vdots & \vdots & \vdots & \vdots & \vdots \\ M_{n-1,1}N & M_{n-1,2}N & \dots & M_{n-1,m-1}N & M_{n-1,m}N \\ M_{n,1}N & M_{n,2}N & \dots & M_{n,m-1}N & M_{n,m}N \end{bmatrix} \tag{A3}$$

where $M_{i,j}$ is the i-th row, j-th column element of M. The unknown x is given by

$$x = \left\{ \text{the } i\text{-th row of } \begin{bmatrix} \Phi & u^1 & u^2 & \dots & u^p \end{bmatrix} \right\}^T \tag{A4}$$

where u^i is the perturbation vector for the i-th experiment for $i = 1, 2, \ldots, p-1, p$. The correction terms, ΔA and Δb, are given by

$$\Delta A = \begin{bmatrix} V^1_{(1..(L-1))} & V^2_{(1..(L-1))} & \cdots & V^{p-1}_{(1..(L-1))} & V^p_{(1..(L-1))} \\ & & 0_{p\times p(L-1)} & & \end{bmatrix}^T, \quad \text{(A5a)}$$

$$\Delta b = \left\{ \text{the } i\text{-th row of } \begin{bmatrix} V^1_{(2..L)} & V^2_{(2..L)} & \cdots & V^{p-1}_{(2..L)} & V^p_{(2..L)} \end{bmatrix} \right\}^T. \quad \text{(A5b)}$$

II. Least Squares and Total Least Squares Solutions

Although the exact values of the correction terms, ΔA and Δb, as given in (A1) are not known, the structure, i.e., how the noise appears in each element, is known. If the unknown term is ignored, then the problem is solved by the least squares method as follows:

$$x_{\text{LS}} = \left(A^T A\right)^{-1} A^T b. \quad \text{(A6)}$$

However, the least squares solution will then in general contain some error bias and have a large root mean square error when ΔA is not equal to zero [1].

To write (A1) in a more compact form, we make the following definitions:

$$C := \begin{bmatrix} A & b \end{bmatrix}, \quad \text{(A7a)}$$

$$\Delta C := \begin{bmatrix} \Delta A & \Delta b \end{bmatrix}. \quad \text{(A7b)}$$

(A1) is written as

$$(C + \Delta C) \begin{bmatrix} x \\ -1 \end{bmatrix} = 0. \quad \text{(A8)}$$

The TLS problem is then posed as follows [2]:

$$\min_{v,x} \|\Delta C\|_F^2 \text{ subject to (A8)} . \quad \text{(A9)}$$

When the smallest singular value of $[A\ b]$ is not repeated, the solution of the TLS problem is given by

$$x_{\text{TLS}} = \left(A^T A - \lambda^2 I\right)^{-1} A^T b \quad \text{(A10)}$$

where λ is the smallest singular value of $[A\ b]$. The case when the smallest singular value is repeated is not considered since then the solution is not unique. Notice that, compared to the least squares solution, the TLS solution has a correction term, λ^2, at the inverse of the matrix. This reduces the bias in the solution, which is caused by the noise in A. The TLS solution can be also computed using the singular value decomposition as follows:

$$\begin{bmatrix} A & b \end{bmatrix} = U \Sigma V^T \quad \text{(A11)}$$

where $U\Sigma V^T$ is the singular value decomposition of the matrix on the left hand side, U and V are unitary matrices and Σ is a diagonal matrix. Note that the singular value decomposition is used to reduce the dimension of the unknown in [3]. Let V be given by

$$V := \begin{bmatrix} \mathbf{v}_1 & \mathbf{v}_2 & \dots & \mathbf{v}_{n+1} & \mathbf{v}_{n+2} \end{bmatrix} \tag{A12}$$

where $\mathbf{v}_i$ is the $(n+2) \times 1$ vector and is the i-th column of the matrix V. Note that n is the dimension of $x(t)$ or the size of the matrix, F. Then, the solution is given by

$$\begin{bmatrix} x_{\text{TLS}} \\ -1 \end{bmatrix} = -\frac{\mathbf{v}_{n+2}}{\nu} \tag{A13}$$

where ν is the last element of $\mathbf{v}_{n+2}$. Numerically, this is a more robust method than computing the inverse of a matrix.

In addition, the TLS solution, (A10) or (A13), can be derived using the singular value decomposition approach. From (A8), we can see that $x = 0$ cannot be a solution unless $b + \Delta b$ equals zero. Excluding this trivial case, for the existence of the solution of (A8), the matrix on the left hand side has to be singular. That is, we are searching for the ΔC with minimum Frobenius norm that makes $C + \Delta C$ singular. From the singular value decomposition of C, (A11), we can see that the ΔC having the smallest Frobenius norm among the matrices satisfies the singular condition is given by $-Cv_{n+2}v_{n+2}^T$, which makes the smallest singular value of C vanish. Substituting $\Delta C = -Cv_{n+2}v_{n+2}^T$ into (A8) and pre-multiplying by C^T gives

$$\left(C^T C - C^T C v_{n+2} v_{n+2}^T\right) \begin{bmatrix} x \\ -1 \end{bmatrix} = \left(C^T C - \lambda^2 v_{n+2} v_{n+2}^T\right) \begin{bmatrix} x \\ -1 \end{bmatrix} = 0. \tag{A14}$$

Hence, (A13) is the solution of (A14). Moreover, because of (A13) we can say that the TLS solution is the solution of the following eigenvalue problem:

$$C^T C \begin{bmatrix} x_{\text{TLS}} \\ -1 \end{bmatrix} = \lambda^2 \begin{bmatrix} x_{\text{TLS}} \\ -1 \end{bmatrix} \tag{A15}$$

where the eigenvalue is given and the corresponding eigenvector is to be obtained. By substituting $C = [A \mid b]$ into (A15) we obtain

$$\begin{bmatrix} A^T A & A^T b \\ b^T A & b^T b \end{bmatrix} \begin{bmatrix} x_{\text{TLS}} \\ -1 \end{bmatrix} = \lambda^2 \begin{bmatrix} x_{\text{TLS}} \\ -1 \end{bmatrix}. \tag{A16}$$

The first row is given by

$$A^T A x_{\text{TLS}} - A^T b = \lambda^2 x_{\text{TLS}}, \tag{A17}$$

and (A10) is obtained.

III. Constrained Total Least Squares Technique

The TLS solution is not optimal when the two noise terms in A and b are correlated, since one of the main assumptions in this method is that the two noise terms are independent of each other. If there is some correlation between them, this knowledge can be used to improve the solution by using the CTLS technique, [1].

In the Jacobian estimation problem, the two noise terms are obviously correlated because A is a function of the noise term from the sampling time k equal to 2 to L and b is a function of the noise term from k equal to 1 to $L-1$. To use the structural information in ΔC, first the minimal set of noise is defined as follows:

$$v = \begin{bmatrix} v_1^T & v_2^T & \dots & v_{L-1}^T & v_L^T \end{bmatrix}^T \in \mathbb{R}^{nL\times 1}. \tag{A18}$$

If v is not white random noise, a whitening process using Cholesky factorization is performed [1]. In this paper v is assumed to be white noise and this whitening process is therefore not necessary. Consider each column of ΔC, i.e.

$$\Delta C = \begin{bmatrix} \underbrace{\Delta C_1 \ \ \Delta C_2 \ \ \dots \ \ \Delta C_n}_{\text{corresponding to } \Phi \in \mathbb{R}^{n\times n}} & \underbrace{\Delta C_{n+1}}_{\text{crsp. to } u \in \mathbb{R}^{n\times 1}} & \underbrace{\Delta C_{n+2}}_{\text{crsp. to } b \in \mathbb{R}^{(L-1)\times 1}} \end{bmatrix} \tag{A19}$$

where ΔC_i is the i-th column vector of ΔC. More specifically

$$\Delta C_i = \begin{bmatrix} {}^i v_1 & {}^i v_2 & \dots & {}^i v_{L-2} & {}^i v_{L-1} \end{bmatrix}^T \tag{A20}$$

where ${}^i v_j$ is the i-th element of v_j for $i = 1, 2, \dots, n-1, n$, $j = 1, 2, \dots, L-2, L-1$, and

$$\Delta C_{n+1} = \mathbf{0}, \tag{A21a}$$

$$\Delta C_{n+2} = \Delta b = \begin{bmatrix} {}^i v_2 & {}^i v_3 & \dots & {}^i v_{L-1} & {}^i v_L \end{bmatrix}^T. \tag{A21b}$$

Each ΔC_i can be written as

$$\Delta C_i = G_i v \tag{A22}$$

for $i = 1, 2, \dots, n+1, n+2$.

To obtain the explicit form for each G_i, we first define the following column vector:

$$e_i = \begin{bmatrix} 0 & 0 & \dots & 0 & \underbrace{1}_{i\text{-th element}} & 0 & \dots & 0 \end{bmatrix}^T \tag{A23}$$

where e_i is $n \times 1$ for $i = 1, 2, \ldots, n-1, n$. For i equal to 1,

$$\begin{aligned}\Delta C_1 &= \begin{bmatrix} {}^1v_1 & {}^1v_2 & \ldots & {}^1v_{L-2} & {}^1v_{L-1}\end{bmatrix}^T \\ &= \begin{bmatrix} v_1^T e_1 & v_2^T e_1 & \ldots & v_{L-2}^T e_1 & v_{L-1}^T e_1\end{bmatrix}^T \\ &= \left\{ v^T \underbrace{\begin{bmatrix} \underbrace{I_{L-1} \otimes e_1}_{n(L-1)\times(L-1)} \\ 0_{n\times(L-1)} \end{bmatrix}}_{nL\times(L-1)} \right\}^T = \underbrace{\begin{bmatrix} \underbrace{(I_{L-1} \otimes e_1)^T}_{(L-1)\times n(L-1)} & 0_{(L-1)\times n} \end{bmatrix}}_{(L-1)\times nL} v.\end{aligned} \tag{A24}$$

Likewise for the i-th column of ΔC

$$\Delta C_i = \begin{bmatrix} (I_{L-1} \otimes e_i)^T & 0_{(L-1)\times n} \end{bmatrix} v, \tag{A25}$$

and hence,

$$G_i = \begin{bmatrix} (I_{L-1} \otimes e_i)^T & 0_{(L-1)\times n} \end{bmatrix} \tag{A26}$$

for $i = 1, 2, \ldots, n-1, n$. Also, from (A21)

$$G_{n+1} = 0_{(L-1)\times nL}, \tag{A27a}$$

$$G_{n+2} = \begin{bmatrix} 0_{(L-1)\times n} & (I_{L-1} \otimes e_i)^T \end{bmatrix}. \tag{A27b}$$

Since ΔC can be written as

$$\Delta C = \begin{bmatrix} G_1 v & G_2 v & \ldots & G_{n+1} v & G_{n+2} v \end{bmatrix}, \tag{A28}$$

then the TLS problem is recast as follows [1]:

$$\min_{v,x} \|v\|^2 \tag{A29}$$

subject to

$$\left\{ C + \begin{bmatrix} G_1 v & G_2 v & \ldots & G_{n+1} v & G_{n+2} v \end{bmatrix} \right\} \begin{bmatrix} x \\ -1 \end{bmatrix} = 0. \tag{A30}$$

This is called the constrained total least squares (CTLS) problem. With the following definition:

$$H_x := \sum_{i=1}^{n} x_i G_i + x_{n+1} G_{n+1} - G_{n+2} = \sum_{i=1}^{n+1} x_i G_i - G_{n+2} \tag{A31}$$

where x_i for $i = 1, 2, \ldots, n-1, n$ is the ith element of Φ for a given row and x_{n+1} is an element in the given row of u, (A30) can be written in the following form:

$$C \begin{bmatrix} x \\ -1 \end{bmatrix} + H_x v = 0. \tag{A32}$$

Solving for v, we get

$$v = -H_x^{\dagger} C \begin{bmatrix} x \\ -1 \end{bmatrix} \tag{A33}$$

where $H^{\dagger}$ is the pseudoinverse of H_x. Hence, the original constrained minimisation problem, (A29), is transformed into an unconstrained minimisation problem as follows:

$$\min_{v,x} \|v\|^2 = \min_{x} \begin{bmatrix} x^T & -1 \end{bmatrix} C^T H_x^{\dagger T} H_x^{\dagger} C \begin{bmatrix} x \\ -1 \end{bmatrix} \tag{A34}$$

Now, we introduce two assumptions, which make the formulation simpler,

- The number of measurements are always strictly greater than the number of unknowns, i.e., we only consider the overdetermined case, explicitly $L > n + 2$. That is, the number of measurements are strictly greater than the dimension of the states plus two. In the notation of [1] the inequality is $M > L$.
- H_x is full rank.

Then, the pseudoinverse is given by

$$H_x^{\dagger} = H_x^T \left(H_x H_x^T \right)^{-1}. \tag{A35}$$

and the non-constrained minimisation problem can be further simplified as follows:

$$\min_{x} \begin{bmatrix} x^T & -1 \end{bmatrix} C^T \left(H_x H_x^T \right)^{-1} C \begin{bmatrix} x \\ -1 \end{bmatrix}. \tag{A36}$$

In [1] the minimisation problem, (A36), is solved using Newton's method. In this paper we simply use either the standard optimisation function in MATLAB, i.e. *fminsearch*, which is a non-constrained optimisation problem solver, or the nonlinear optimisation function from the MATLAB optimization toolbox, i.e. *fmincon*, which is a constrained optimisation problem solver. The initial condition for x used in the optimisation algorithms is simply that returned by the solution of the standard least squares problem.

If p experiments are performed around the same equilibrium point, the matrices G_i for $i = 1, 2, \ldots, n+1, n+2$ are formulated from (A5). The dimension of the minimal set of noise increases as follows:

$$v = \begin{bmatrix} \left(v^1\right)^T & \left(v^2\right)^T & \ldots & \left(v^{p-1}\right)^T & \left(v^p\right)^T \end{bmatrix}^T \in \mathbb{R}^{pnL \times 1} \tag{A37}$$

where v^i for $i = 1, 2, \ldots, p-1, p$ is the noise vector, given in (A18), for one experiment. In addition ΔC is given by

$$\Delta C = \begin{bmatrix} \underbrace{\Delta C_1 \;\; \Delta C_2 \;\; \ldots \;\; \Delta C_n}_{\text{crsp. to } \Phi} & \underbrace{\Delta C_{n+1}}_{\text{crsp. to } u^1} & \underbrace{\Delta C_{n+2}}_{\text{crsp. to } u^2} & \cdots & \underbrace{\Delta C_{n+p}}_{\text{crsp. to } u^p} & \underbrace{\Delta C_{n+p+1}}_{\text{crsp. to } b} \end{bmatrix}. \tag{A38}$$

Consider the first column of ΔC

$$\begin{aligned}
\underbrace{\Delta C_1}_{p(L-1)\times 1} &= \left\{ \left(v^1\right)^T \begin{bmatrix} I_{L-1}\otimes e_1 \\ 0_{n\times(L-1)} \end{bmatrix},\ \left(v^2\right)^T \begin{bmatrix} I_{L-1}\otimes e_1 \\ 0_{n\times(L-1)} \end{bmatrix},\ \ldots,\ \left(v^p\right)^T \begin{bmatrix} I_{L-1}\otimes e_1 \\ 0_{n\times(L-1)} \end{bmatrix} \right\}^T \\
&= \left[\left(v^1\right)^T G_1^T,\ \ \left(v^2\right)^T G_1^T,\ \ \ldots,\ \ \left(v^p\right)^T G_1^T \right]^T \\
&= \left\{ \begin{bmatrix} G_1^T \\ 0_{nL(p-1)\times(L-1)} \end{bmatrix},\ \begin{bmatrix} 0_{nL\times(L-1)} \\ G_1^T \\ 0_{nL(p-2)\times(L-1)} \end{bmatrix},\ \begin{bmatrix} 0_{2nL\times(L-1)} \\ G_1^T \\ 0_{nL(p-3)\times(L-1)} \end{bmatrix},\ \ldots,\ \begin{bmatrix} 0_{nL(p-1)\times(L-1)} \\ G_1^T \end{bmatrix} \right\}^T v \\
&= \left[I_p \otimes G_1 \right] v,
\end{aligned} \tag{A39}$$

hence,

$$\mathbf{G}_i = \left[I_p \otimes G_i \right], \tag{A40}$$

for $i = 1,\ 2,\ \ldots,\ n-1,\ n$ where bold face type denotes the quantity in the case of multiple experiments. Also,

$$\mathbf{G}_i = 0_{p(L-1)\times pnL} \tag{A41}$$

for $i = n+1,\ n+2,\ \ldots,\ n+p-1,\ n+p$. Now, consider the last column of ΔC

$$\begin{aligned}
\Delta C_{n+p+1} &= \left\{ \left(v^1\right)^T \begin{bmatrix} 0_{n\times(L-1)} \\ I_{L-1}\otimes e_j \end{bmatrix},\ \left(v^2\right)^T \begin{bmatrix} 0_{n\times(L-1)} \\ I_{L-1}\otimes e_j \end{bmatrix},\ \ldots,\ \left(v^p\right)^T \begin{bmatrix} 0_{n\times(L-1)} \\ I_{L-1}\otimes e_j \end{bmatrix} \right\}^T \\
&= \left[\left(v^1\right)^T G_{n+2}^T,\ \ \left(v^2\right)^T G_{n+2}^T,\ \ \ldots,\ \ \left(v^p\right)^T G_{n+2}^T \right]^T \\
&= \left\{ \underbrace{v^T}_{1\times pnL} \underbrace{\begin{bmatrix} G_{n+2}^T \\ 0_{nL\times(L-1)} \\ 0_{nL\times(L-1)} \\ \vdots \\ 0_{nL\times(L-1)} \end{bmatrix}}_{pnL\times(L-1)},\ v^T \begin{bmatrix} 0_{nL\times(L-1)} \\ G_{n+2}^T \\ 0_{nL\times(L-1)} \\ \vdots \\ 0_{nL\times(L-1)} \end{bmatrix},\ \ldots,\ v^T \begin{bmatrix} 0_{nL\times(L-1)} \\ 0_{nL\times(L-1)} \\ \vdots \\ 0_{nL\times(L-1)} \\ G_{n+2}^T \end{bmatrix} \right\}^T \\
&= \left[I_p \otimes G_{n+2} \right] v.
\end{aligned} \tag{A42}$$

Hence,

$$\mathbf{G}_{n+2} = \left[I_p \otimes G_{n+2} \right]. \tag{A43}$$

Note that the size of the matrices increases very fast with the number of data points and the number of experiments. However, most of the terms in the matrices are zero, i.e. they are sparse matrices. In this case, the required amount of computer memory and calculation time increases more slowly with the dimension of the matrices - in MATLAB there is a command *sparse* to treat sparse matrices efficiently.

References

[1] T. J. Abatzoglou, J. M. Mendel, and G. A. Harada, "The constrained total least squares technique and its application to harmonic superresolution," *IEEE Transactions on Signal Processing*, vol. 39, no. 5, pp. 1070–1087, May 1991.

[2] G. H. Golub and C. F. V. Loan, "An analysis of the total least squares problem," *SIAM Journal on Numerical Analysis*, vol. 17, no. 6, pp. 883–893, December 1980.

[3] M. Bansal, G. D. Gatta, and D. di Bernardo, "Inference of gene regulatory networks and compound mode of action from time course gene expression profiles," *Bioinformatics*, vol. 22, no. 7, pp. 815–822, April 2006.
